# Exploiting nonlinearities through geometric engineering to enhance the auxetic behaviour in re-entrant honeycomb metamaterials

**DOI:** 10.1038/s41598-023-47525-7

**Published:** 2023-11-27

**Authors:** Chetna Srivastava, Lalit Bhola, Vinyas Mahesh, P. J. Guruprasad, Nik Petrinic, Fabrizio Scarpa, Dineshkumar Harursampath, Sathiskumar A. Ponnusami

**Affiliations:** 1 NMCAD Lab, Department of Aerospace Engineering, Indian Institute of Science, Bengaluru, 560012 India; 2 Department of Aerospace Engineering, Indian Institute of Technology Bombay, Mumbai, 400076 India; 3 Department of Engineering, City, University of London, Northampton Square, London, EC1V 0HB UK; 4 Department of Engineering Science, University of Oxford, Parks Road, Oxford, OX1 3PJ Oxfordshire UK; 5 Bristol Composites Institute, University of Bristol, Bristol, BS8 1TR UK

**Keywords:** Mechanical engineering, Aerospace engineering

## Abstract

Classical approaches to enhance auxeticity quite often involve exploring or designing newer architectures. In this work, simple geometrical features at the member level are engineered to exploit non-classical nonlinearities and improve the auxetic behaviour. The structural elements of the auxetic unit cell are here represented by thin strip-like beams, or thin-walled tubular beams. The resulting nonlinear stiffness enhances the auxeticity of the lattices, especially under large deformations. To quantify the influence of the proposed structural features on the resulting Poisson’s ratio, we use here variational asymptotic method (VAM) and geometrically exact beam theory. The numerical examples reveal that 2D re-entrant type micro-structures made of thin strips exhibit an improvement in terms of auxetic behaviour under compression. For the auxetic unit cell with thin circular tubes as members, Brazier’s effect associated with cross-sectional ovalisation improves the auxetic behaviour under tension; the enhancement is even more significant for the 3D re-entrant geometry. Thin strip-based auxetic unit cells were additively manufactured and tested under compression to verify the numerical observations. The experimentally measured values of the negative Poisson’s ratio are in close agreement with the numerical results, revealing a 66% increase due to the nonlinearity. Simulation results showcase these alternative approaches to improve the auxetic behaviour through simple geometric engineering of the lattice ribs.

## Introduction

A negative value of Poisson’s ratio is physically counterintuitive for traditional engineering materials because a longitudinal extension is coupled with a contraction along the transverse/lateral direction in the case of conventional materials. Auxetic metamaterials are designed with this unique mechanical characteristic^1^. The peculiarity in their mechanical behaviour is attributed to the deformation mechanism of their microstructure. With recent advancements in additive manufacturing^2^, different geometries of unit cells showing auxetic behaviour have been the subject of various analytical, numerical as well as experimental investigations^3–8^.

The majority of the deformation mechanisms of auxetic metamaterials can be categorized into three major groups: the re-entrant, chiral and rotating rigid units. Since auxetic materials have been found to exhibit better indentation resistance, impact absorption and damage tolerance^9–13^, the design of micro-structure geometries with a negative value of Poisson’s ratio have been the focus of a number of pioneering works^14– 22^. Design of new architectures has been found to be the central framework in important works related to the design of architectured materials with desired properties^23^. For example, Fu et al.^24^ presented a novel 3D geometry of the microstructure, wherein neighbouring layers of tetrachiral honeycombs were interconnected by inclined rods. Javadi et al.^25^ used a combination of finite element method and genetic algorithm to design geometries with a given value of Poisson’s ratio as a constraint, while Wang et al.^26^ performed design optimizations considering stiffness as a constraint to determine the minimum Poisson’s ratio attainable for tetra-petal auxetic structures. Harkati et al.^27^ presented a new auxetic honeycomb configuration with curved cell walls, while Zhang et al.^28^ designed metamaterials with re-entrant geometry and star-shaped nested cells. In addition to topology optimization, foams exhibiting auxetic behaviour have also been fabricated^19,29,30^.

The characterization of auxetic materials involves determination of the effective elastic properties, i.e., Young’s modulus and Poisson’s ratio. Closed-form expressions for effective elastic properties in terms of geometric and material parameters have been previously derived by discretizing the geometry into beams and imposing suitable boundary conditions. Within the framework of small deformation theories such as the Euler–Bernoulli beam model^31–34^, these expressions are independent of applied strain and stress. The properties of auxetic materials have previously also been determined using strain-based homogenization methods within the framework of linear elasticity^35– 37^. A number of studies have been focused on the design of metamaterials using inverse homogenization, wherein an optimization problem is formulated to design materials with a target value of homogenized elastic coefficients^38,39^.

There have been attempts to predict the mechanical behavior of auxetic honeycombs under large far-field stresses and the related non-linearity of their equivalent material behaviour using large deformation beam theories. Previously for the re-entrant geometry, the elastica theory has been used to represent the behaviour of the lattice beams; in that case, geometrically exact expressions for the curvature are adopted for the equilibrium equations to determine the variation of the Poisson’s ratio with applied strains^40,41^. Gao et al.^42^ have used a similar methodology to determine strain-dependent mechanical behaviour for the double V geometry of the microstructure. Zhang et al.^43^ studied the behaviour of a series of auxetic geometries under large deformations, particularly focusing on their energy absorption characteristics. Hu^44^ has identified the collapse behaviour of re-entrant antitetrachiral honeycombs under large deformations, while Jianga et al.^45^ have determined the limiting strains for the auxetic behaviour in chiral and re-entrant geometries under large strains.

Most of the research reported in the open literature and related to using large deformation unit cell member models is limited to the analysis of the strain-dependent behaviour of the auxetic geometries. To the best of the authors’ knowledge, the one-dimensional non-linearity along the beam reference line or large deflections of the members constituting the auxetic frame has not been taken so far into account for shape optimization and topology-based design. In addition to the one-dimensional non-linearity attributed to large displacements and rotations, specific non-linear effects arise due to excessive warping of the cross-section, which are termed as non-classical non-linearities. Some well-known examples of these types of nonlinearities include the trapeze^46^ and the Brazier’s effect^47,48^. The trapeze effect is a phenomenon wherein an extension-twist coupling arises in thin strips. The Brazier’s effect is characterized by a marked reduction of the bending stiffness of thin circular tubes with increasing magnitudes of the curvature.

In contrast to previous works wherein the improvement of the auxetic characteristics was driven by the design of new unit cell topologies, we propose here to exploit the non-classical geometric nonlinearities arising due to cross-sectional warping^46,47^. To assess the effectiveness of using this approach, we modify the ribs of 2D and 3D re-entrant lattice metamaterials topologies to undergo the non-classical nonlinear behaviour and investigate the auxeticity of the metamaterials under large deformations.

In this paper, the methodology, the mathematical formulations and the geometrical configurations are described in "Numerical methodology". The experimental validation for the numerical methodology is summarised in "Experimental methodology". Load versus Poisson’s ratios for the 2D and 3D re-entrant geometries and their parametric evaluations are presented in "Results and discussions", followed by conclusions in "Conclusions".

## Numerical methodology

The microstructure configurations of the 2D and 3D re-entrant geometries have been discretized into beams with boundary conditions as per Wan et al.^40^ and Yang et al.^49^. The beams have cross-sections typical of thin strips and thin circular tubes. A parametric analysis has been carried out for different values of the model parameters, i.e., the rib-inclination angles, thickness-to-width ratios for thin strips and thickness-to-radius ratios for thin circular tubes, i.e., θ, t/b, and t/R respectively.

**Figure 1:**
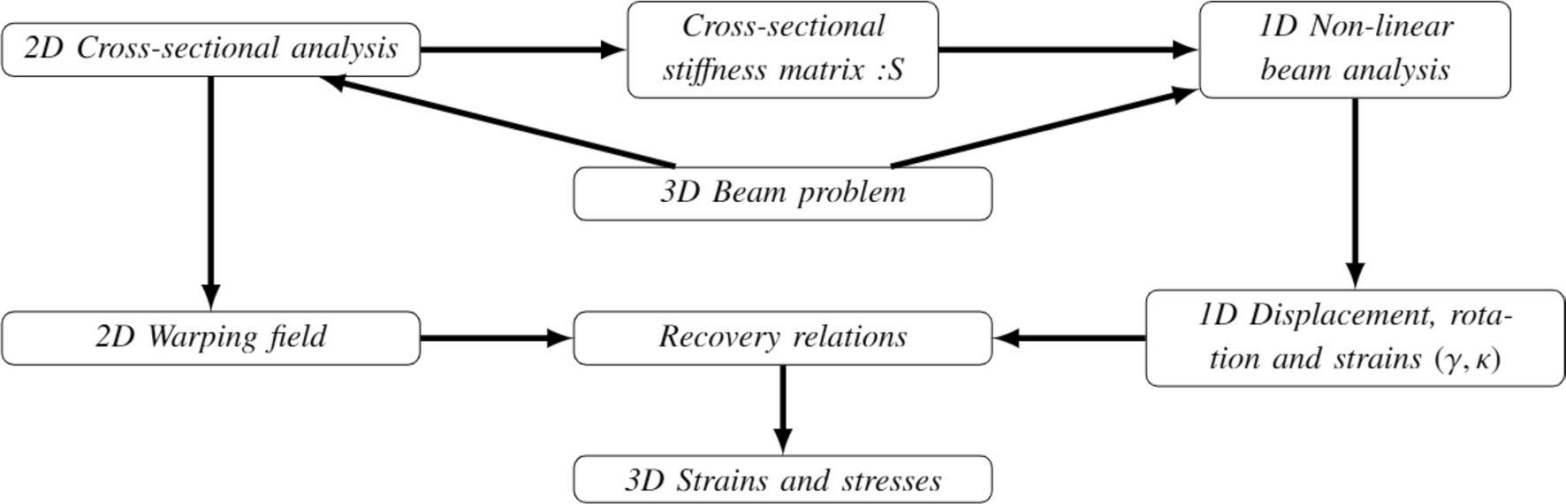
Methodology for beam analysis adopted in this work (ref. Hodges et al.^50,51^).

To account for the non-classical effects, the one-dimensional non-linear beam analysis should be coupled with a two-dimensional non-linear cross-section analysis. In contrast, conventional beam theories neglect the cross-sectional deformations or tend to impose certain kinematic assumptions, while reducing the actual three-dimensional beam to a one-dimensional problem. Hodges^50,51^ decoupled the original three-dimensional beam problem into two analyses, one related to a two-dimensional cross-section and the other to a one-dimensional non-linear beam, as shown in Fig. 1. This methodology accurately captures both the in-plane and out-of-plane warping of the cross-section. As shown in Fig. 1, the cross-sectional stiffness matrix obtained from the two-dimensional analysis is a required input for the one-dimensional non-linear beam problem. As non-classical nonlinear effects become significant, the stiffness coefficients would vary as a function of the one-dimensional strains along the beam reference line. This leads to a double nonlinear problem, i.e., nonlinear both along the beam reference line and over the cross-section.

Previously, the methodology for determining the nonlinear stiffness coefficients in terms of one-dimensional strain measures for thin strips^46^, as well as for circular tubes^47^ has been developed. This method involves the identification of small parameters associated with problem geometry, and the minimization of strain energy w.r.t. the warping field after eliminating higher order terms from the functional using variational asymptotic method. The cross-sectional stiffness coefficients for thin strips and circular tubes have been adopted from^46,47^, whereby the nonlinear stiffness coefficients are derived for an isotropic material in this work.

**Figure 2:**
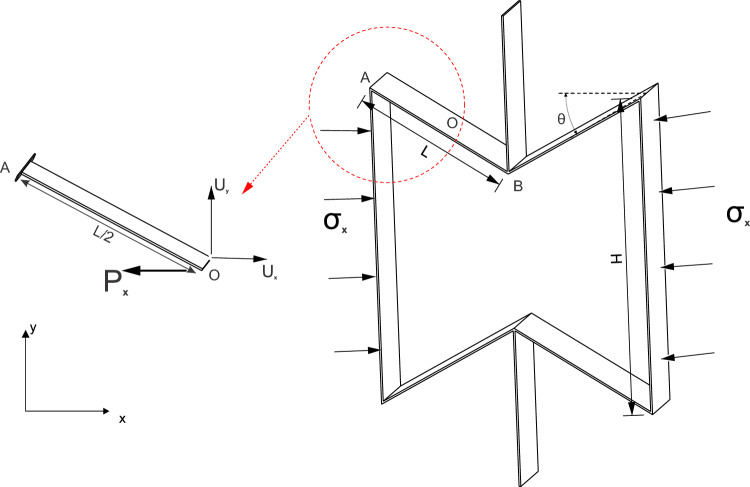
2D re-entrant geometry with the inclination angle of the ribs θ, the length of inclined member $L$ and height of vertical member $H$. Half-length cantilever beam model for the 2D re-entrant geometry^40^: the inclined member AB is split into two half-length cantilever beams, i.e., $AO$ and $OB$. The tip deflections in the horizontal and vertical directions for the cantilever beam are ${U}_{x}$ and ${U}_{y}$, respectively. The tip load on the cantilever beam is determined from the applied far-field stress, i.e., ${\sigma }_{x}$.

In the case of re-entrant geometry, the hinging of thin buckled cell ribs provides the negative value of the Poisson’s ratio of re-entrant honeycombs at the macroscale. Wan et al.^40^ and Levy and Goldfarb^41^ used therefore a large deflection model based on the elastica theory to determine the effective value of the Poisson’s ratio under large far-field stresses by considering only the deformation of the inclined member AB in Fig. 2. Since the unit cell is part of a larger continuum, the symmetry of the deformation is imposed by suppressing the rotations at the joints A and B, thereby implying that the inclined beam AB can be broken into two half cantilever beams (Fig. 2).

The tip deflection for the cantilever beams along the horizontal and vertical directions ${U}_{x}$ and ${U}_{y}$ under the applied load ${P}_{x}$ are determined for the different values of the rib-inclination angles $\theta$. The Poisson’s ratio is evaluated as per Eq. (1), wherein $Lcos\theta$ and $(H-Lsin\theta )$ are the horizontal and vertical projections respectively of the undeformed beam:

1 $${v}_{yx}=-\frac{{\epsilon }_{y}}{{\epsilon }_{x}}=-\frac{{U}_{y}Lcos\theta }{{U}_{x}\left(H-Lsin\theta \right)}$$

The definition of Poisson’s ratio is instantaneous and not incremental as indicated in Eq. (1). Within the framework of the linear beam theories for small deflections, the value of the Poisson’s ratio is independent of the applied stress or strain field, and Eq. (1), is reduced to the following form:

2 $${v}_{yx}=\frac{(3-{k}^{2})sin\theta }{[\alpha -sin\theta ][3+{k}^{2}{tan}^{2}\theta ]}$$

**Figure 3:**
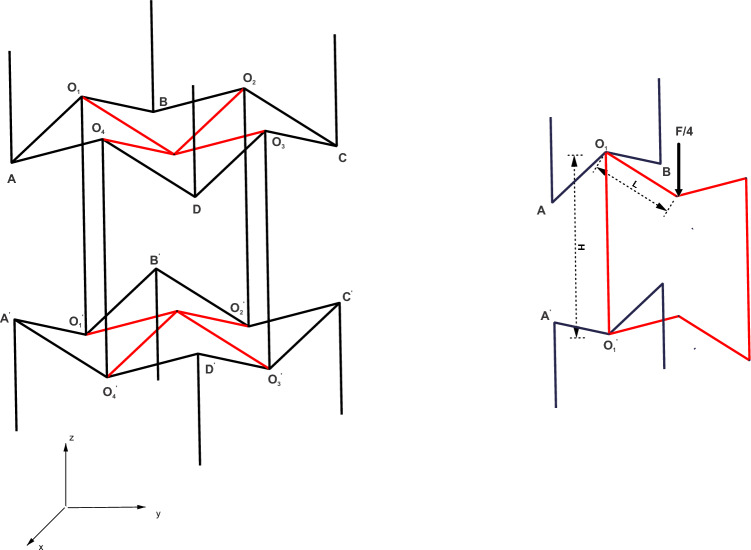
3D re-entrant geometry with the rib-inclination angle θ, length of inclined member $L$ and height of vertical member $H$. The half-length cantilever beam model for the 3D re-entrant geometry^49^: the inclined member is split into two half-length cantilever beams. The tip deflections in the horizontal and vertical directions for the cantilever beam are $\Delta { x}_{1}$ and $\Delta { z}_{1}$, respectively. The tip load on the cantilever beam is determined from the applied far-field stress ${\sigma }_{z}$.

In Eq. (2), $k$ is the slenderness ratio of the inclined member AB and α is the ratio of the length of the vertical strut to the inclined strut, i.e., $H/L$. Similarly, Yang et al.^49^ formulated analytical expressions for the Poisson’s ratio and the effective Young’s modulus for the 3D re-entrant geometry shown in Fig. 3. Under the effect of applied far-field stresses in the vertical z direction, the inclined members deform symmetrically. Hence, from symmetry considerations, Yang et al.^49^ determined the value of the Poisson’s ratio from the deformed configuration of the inclined member ${O}_{1}B$ and the vertical member ${O}_{1}{O'}_{1}$ as shown in Fig. 3, while restricting the rotations at the joint ${O}_{1}$. The value of the Poisson’s ratio in the z direction, i.e., ${v}_{xz}$ for the three-dimensional geometry has been determined from the deformations of the inclined and vertical struts as per the following equation:

3 $${v}_{xz}=-\frac{2\Delta {x}_{1}(H-Lcos\theta )}{(2\Delta {z}_{1}+\Delta {z}_{2})Lsin\theta }$$

where, H, L and θ are the model parameters shown in Fig. 3, $\Delta {x}_{1}$ and $\Delta {z}_{1}$ are the horizontal and vertical tip deflections for the half-length cantilever beam representing the inclined members of length L, and $\Delta {z}_{2}$ is the vertical deflection for the member ${O}_{1}{O'}_{1}$. When using small deformations, Eq. (3) is reduced to the following form, wherein $\Delta {z}_{2}$ is neglected:

4 $${v}_{xz}=\frac{(12-{k}^{2})cos\theta (\alpha -cos\theta )}{[{k}^{2}{sin}^{2}\theta +12{cos}^{2}\theta ]}$$

In Eq. (4), $k$ is the slenderness ratio of the inclined member ${O}_{1}{B}$. For slender struts, the Poisson’s ratio for two-dimensional re-entrant geometry was shown to vary significantly with the applied stress^40^. The deviation from the strain-independent value of Poisson’s ratio determined as per Eq. (2) was primarily attributed to large deformations, which have been previously accounted for using the elastica theory by Wan et al.^40^ and Levy and Goldfarb^41^, however, the results were obtained considering physically unrealistic values of tip deflection angles as inputs. In contrast to formulations previously presented, the geometrically-exact beam theory presented in “Geometrically exact beam theory” is used here to determine the deflections of the struts under large loads.

### Cross-sectional analysis

The mathematical formulations in the following sections have been adopted from the asymptotic theory presented in previous works^46,47^. The asymptotic theory has been adapted to thin structural members for the auxetic configurations in Figs. 2 and 3. The material for the frame is assumed to be linear elastic and isotropic and the members constituting the frame are assumed to be thin strips (“Strip-based structural member”) and circular tubes (“Tubular geometry”):

#### Strip-based structural member:

**Figure 4:**
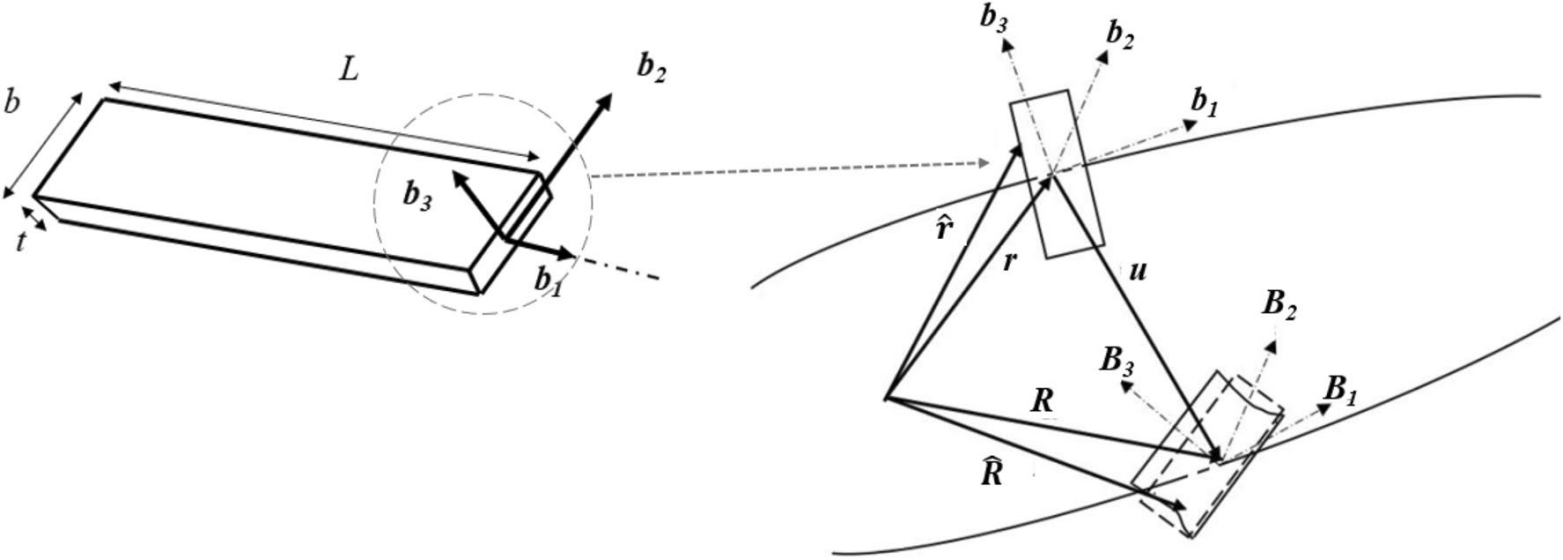
Thin strip with geometric parameters: length of member $L$, width $b$ and thickness $t$: Undeformed and deformed configurations of the cross-section, position vector to a material point on the undeformed cross-section, i.e.$\widehat{{\varvec{r}}}$ is expressed in terms of measure numbers along the orthonormal triad of ${{\varvec{b}}}_{{\varvec{i}}}$ vectors, and the position vector to a material point on the deformed cross-section, i.e. $\widehat{{\varvec{R}}}$ is expressed in terms of measure numbers along the orthonormal triad of ${{\varvec{B}}}_{{\varvec{i}}}$ vectors.

A strip is a thin rectangular member for which the thickness is significantly smaller than the width, as shown in Fig. 4. As indicated in Fig. 4, the length of the beam is $L$, the width of the strip is $b$ and the thickness of the strip is $t$. The variational method involves constrained minimization of the strain energy functional. Smallness of certain parameters allows for the elimination of higher-order terms in the energy with respect to these parameters. A preliminary analysis is performed considering only the zeroth order terms, followed by a more refined analysis taking into consideration the first order terms as well. For each strip, the small parameters which can be clearly identified are the thickness-to-width ratio, i.e., ${\delta }_{t}=\left(\frac{t}{b}\right)$ , width to length ratio, i.e., ${\delta }_{b}=\left(\frac{b}{L}\right)$ and width times pretwist per unit length, i.e., ${\delta }_{k}=a{k}_{1}.$

Equation (5) is the expression for one-dimensional strain energy, i.e., ${U}_{1D}$, in terms of the one-dimensional strains along the beam reference line, i.e., extension, twist, and bending curvature, i.e., ${\Upsilon }_{11}$, ${\kappa }_{1}$, and ${\kappa }_{2}$/${\kappa }_{3}$ respectively, wherein ${S}_{l}$, ${S}_{ln}$ and ${S}_{n}$ are the coefficient matrices (ref. Eqs. A.9, A.10 and A.11). The details of the mathematical formulations for the strain energy have been summarised in “Appendix A”^46^.

5 $${U}_{1D} =\frac{1}{2}{\epsilon }_{l}^{T}\left[{S}_{l}\right]{\epsilon }_{l}+{\epsilon }_{l}^{T}\left[{S}_{ln}\right]{\epsilon }_{n}+\frac{1}{2}{\epsilon }_{n}^{T}\left[{S}_{n}\right]{\epsilon }_{n}$$

The linear and non-linear terms in the expression for one-dimensional strain energy can be combined to obtain the equivalent stiffness matrix, i.e.:

$${U}_{1D} =\frac{1}{2}{\epsilon }_{l}^{T}\left[{S}_{eq}\right]{\epsilon }_{l}$$

where, for an isotropic material with Young’s modulus E, Poisson’s ratio ν, and pretwist along the reference line ${k}_{1}=0$, equivalent stiffness matrix, ${S}_{eq}$ is:

$${S}_{eq} = \left[\begin{array}{cccc}Ebt& \frac{E{b}^{3}t}{24}{\kappa }_{1}& 0& 0\\ \frac{E{b}^{3}t}{24}{\kappa }_{1}& \frac{E{b}^{3}t}{6\left(1+\upnu \right)}+\frac{E{b}^{5}t}{320}{\kappa }_{1}^{2}& 0& 0\\ 0& 0& \frac{Eb{t}^{3}}{12}+\frac{E{b}^{5}t{v}^{2}}{720}{\kappa }_{2}^{2}+\frac{E{b}^{5}tv}{360}{\kappa }_{1}^{2}-\frac{E{b}^{7}}{2520t}{\kappa }_{3}^{2}& 0\\ 0& 0& 0& \frac{E{b}^{3}t}{12}\end{array}\right]$$

In accordance with the flow chart presented in Fig. 1, the cross-sectional stiffness matrix ${S}_{eq}$ is a required input for one-dimensional nonlinear beam analysis. However, since the terms of the stiffness matrix are functions of one-dimensional moment strains along the beam reference line, the stiffness matrix is updated after each load increment. Further, from the equivalent cross-sectional matrix obtained, we can infer that the second diagonal term which corresponds to torsional rigidity increases as the moment strain ${\kappa }_{1}$ increases with or without pre-twist. This phenomenon is recognized as the well-known non-physical nonlinearity, i.e., the so-called trapeze effect. In addition, the third diagonal term, corresponding to bending stiffness increases as well with increasing moment strains, i.e., ${\kappa }_{2}$. The effect of the nonlinearity arising due to the smallness of the parameter ${\delta }_{t}$ can be suppressed by replacing ${S}_{eq}$ with ${S}_{l}$, for which the stiffness coefficients are constants.

Additionally, the zeroth order solution for shell out of plane warping (along ${{\varvec{b}}}_{{\varvec{3}}}$ direction in Fig. 4) of thin isotropic strip from^46^ for (${\kappa }_{1}$ = 0, ${\kappa }_{3}$ = 0) is of the following form when bending, i.e., ${\kappa }_{2}$ is predominant:

6 $${w}_{3}^{o}=\frac{\left(12{x}_{2}^{2}-{b}^{2}\right)v{\kappa }_{2}}{24}$$

where, ${x}_{2}$ is measured along the width direction for the thin strip (i.e. along ***b***_***2***_ direction). From Eq. (6), it can be inferred that the deformed shape of the cross-section would be parabolic.

#### Tubular geometry

**Figure 5:**
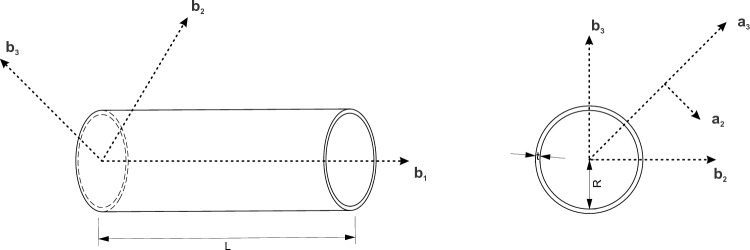
Thin circular tube with geometric parameters: length $L$, radius $R$ and thickness $t$. Position vector to a material point on the undeformed cross-section, i.e., $\widehat{{\varvec{r}}}$ is expressed in terms of measure numbers along the orthonormal triad of ${{\varvec{b}}}_{{\varvec{i}}}$ vectors. For the curvilinear coordinate system is ${{\varvec{a}}}_{1}$ coincides with ${{\varvec{b}}}_{1}$ and ${{\varvec{a}}}_{2}$ and ${{\varvec{a}}}_{3}$ are along the tangential and radial directions respectively.

A thin circular tube is shown in Fig. 5 for which the small parameters that can be identified are the ratio of thickness to the radius, i.e., ${\delta }_{h}=\left(\frac{t}{R}\right)$ and the ratio of radius to the length of the tube, i.e., ${\delta }_{R}=\left(\frac{R}{L}\right)$. The one-dimensional strain energy obtained after integration along the circumference of the tube is expressed as follows (ref. “Appendix B”):

7 $${U}_{1D} =\frac{1}{2}\left[\begin{array}{cccc}{\Upsilon }_{11}& {\kappa }_{1}& {\kappa }_{2}& {\kappa }_{3}\end{array}\right]\left[\begin{array}{cccc}2\pi Rt& 0& 0& 0\\ 0& \frac{Et\pi {R}^{3}}{\left(1+v\right)}& 0& 0\\ 0& 0& {S}_{22}& 0\\ 0& 0& 0& {S}_{33}\end{array}\right]\left[\begin{array}{c}{\Upsilon }_{11}\\ {\kappa }_{1}\\ {\kappa }_{2}\\ {\kappa }_{3}\end{array}\right]$$

where

8 $${S}_{22}=\frac{\pi {R}^{3}Et}{(1-{v}^{2})}\bigg[1-\frac{9(R{\kappa }_{2}{)}^{2}}{12(t/R{)}^{2}+10(R{\kappa }_{2}{)}^{2}}\bigg]$$

9 $${ S}_{33}=\frac{\pi {R}^{3}Et}{(1-{v}^{2})}\bigg[1-\frac{9(R{\kappa }_{3}{)}^{2}}{12(t/R{)}^{2}+10(R{\kappa }_{3}{)}^{2}}\bigg]$$

Hence, the cross-sectional stiffness matrix, ${S }_{eq}$ is obtained to be of the following form:

10 $$S_{eq}=\left[\begin{array}{cccc}2\pi Rt& 0& 0& 0\\ 0& \frac{Et\pi {R}^{3}}{\left(1+v\right)}& 0& 0\\ 0& 0& \frac{\pi {R}^{3}Et}{\left(1-{v}^{2}\right)}\left[1-\frac{9(R{\upkappa }_{2}{)}^{2}}{12(t/R{)}^{2}+10(R{\upkappa }_{2}{)}^{2}}\right]& 0\\ 0& 0& 0& \frac{\pi {R}^{3}Et}{\left(1-{v}^{2}\right)}\left[1-\frac{9(R{\upkappa }_{3}{)}^{2}}{12(t/R{)}^{2}+10(R{\upkappa }_{3}{)}^{2}}\right]\end{array}\right]$$

From the stiffness matrix in Eq. (10), one can infer that the diagonal terms corresponding to moment strain ${\kappa }_{2}$ and ${\kappa }_{3}$ decrease as the magnitude of the curvatures increases. This phenomenon is known as Brazier’s effect, which is a well-known non-physical non-linearity.

### Geometrically exact beam theory

**Figure 6:**
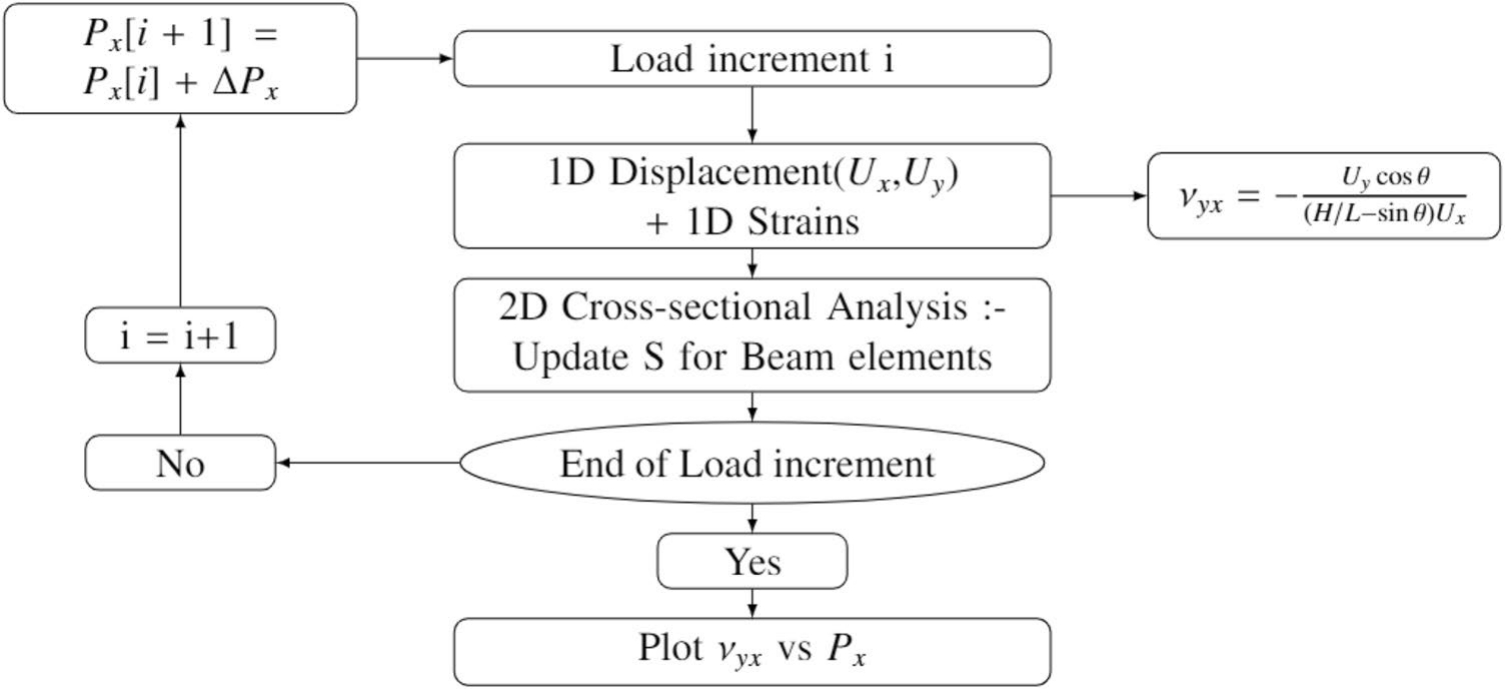
Nonlinear beam analysis procedure.

The cross-sectional nonlinear stiffness matrices obtained in "Cross-sectional analysis" are used as inputs for the nonlinear one-dimensional analysis along the beam reference line. The equations for one-dimensional beam analysis are derived from extended Hamilton’s principle^52^:

11 $${\int }_{{t}_{1}}^{{t}_{2}}{\int }_{0}^{L}[\delta \left(T-U\right)+\delta W]=\delta A$$

where T is the kinetic energy per unit length, U is the strain energy per unit length, W is the virtual work done by the applied loads per unit length, and A is the virtual action at the ends of the beam. For a static problem considered here, the kinetic energy term is eliminated. The mathematical formulations for the Euler–Lagrange equations and the finite element formulation have been summarised in “Appendix C”. The one-dimensional strains determined from Eq. C.17 at a given load increment are used to determine the updated cross-sectional stiffness matrix in “Strip-based structural member” and “Tubular geometry” for the next load step. The iterative nonlinear analysis procedure is summarised in Fig. 6.

## Experimental methodology

In order to validate the behaviour of the geometry as predicted by the numerical methodology (Fig. 6), the 2D re-entrant configurations made of thin strips were fabricated and tested. The procedure for the specimen preparation, test procedure and test response has been summarised in "Specimen geometry and preparation", "Quasi-steady compressive test for auxetic unit cells" and "Quasi-steady compression test response" respectively. It is emphasised here that the objective of the current work is not to conduct comprehensive experimental evaluation, but rather verify the proposed ideas and the numerical results by testing one of the selected auxetic configurations.

The experimental validation was limited to thin strips due to certain limitations of the multi-step additive manufacturing process available for hollow tubular member (ref. “Tubular geometry”). Single-step additive manufacturing techniques are primarily used to fabricate hollow-walled polymeric lattices. In contrast, multi-step additive manufacturing approaches were predominantly used to fabricate metal and ceramic hollow-walled lattices. Manufacturing difficulties regarding geometric precision were reported for additive manufacturing of hollow-walled auxetics. Additive manufacturing of such tubular structure would lead into warping and dislocation during the fabrication process. Mitigating these issues would necessitate the incorporation of reinforcing materials, as reported in^53^. A recent review article discusses the characteristics of hollow-walled lattices while highlighting the manufacturing difficulties and their unique deformation characteristics^54^. A section is devoted in this review article^54^ to the ovalisation of hollow-walled lattices, i.e., Brazier’s effect (“Tubular geometry”) and suggests further research.

### Specimen geometry and preparation

**Figure 7:**
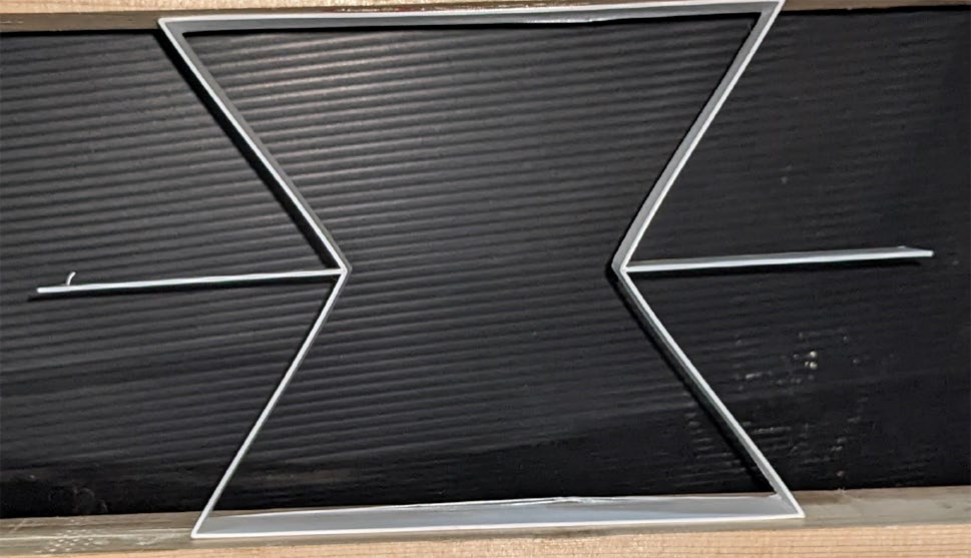
3D printed specimen.

The 3D printed specimen is shown in Fig. 7 and the geometric parameters for the unit cell are listed in Table 1. The material used for printing the specimen is ABS plastic for which the elastic modulus, i.e., E = 2.2 GPa and the Poisson’s ratio is ν = 0.37^ 55^.

**Table 1:** Geometric parameters.

Geometric parameters				
$\theta {(}^{\circ})$	*H* (mm)	*L* (mm)	*b* (mm)	*t* (mm)
$30$	160	80	20	0.6

A 3D printing technique based on the fused deposition method (FDM) is used to fabricate the auxetic unit cell specimens. The FDM printer used is a Flash Forge Guider II printer with ABS filament. The printing parameters and filament material were kept constant to reduce the deviation in the fabricated specimens. The printer setting for the fabrication process is provided in Table 2.

**Table 2:** Printer settings considered for the re-entrant auxetic structure for experiments.

Parameters	Filament diameter	Nozzle diameter	Nozzle temperature	Printing speed	Layer height	Infill density
Specifications	1.75 mm	0.3 mm	240 °C	50 mm/s	0.16	100%

The printed specimens were cleaned through hot air jet cleaning to remove excess material ribbons during the printing process. A digital vernier caliper was used to measure the dimensions of the fabricated sample and the dimensions were compared with the designed structure with dimensions provided in Table 1. The dimension was the same as the designed geometry, which indicated the accuracy of the 3D printer.

### Quasi-steady compressive test for auxetic unit cells

**Figure 8:**
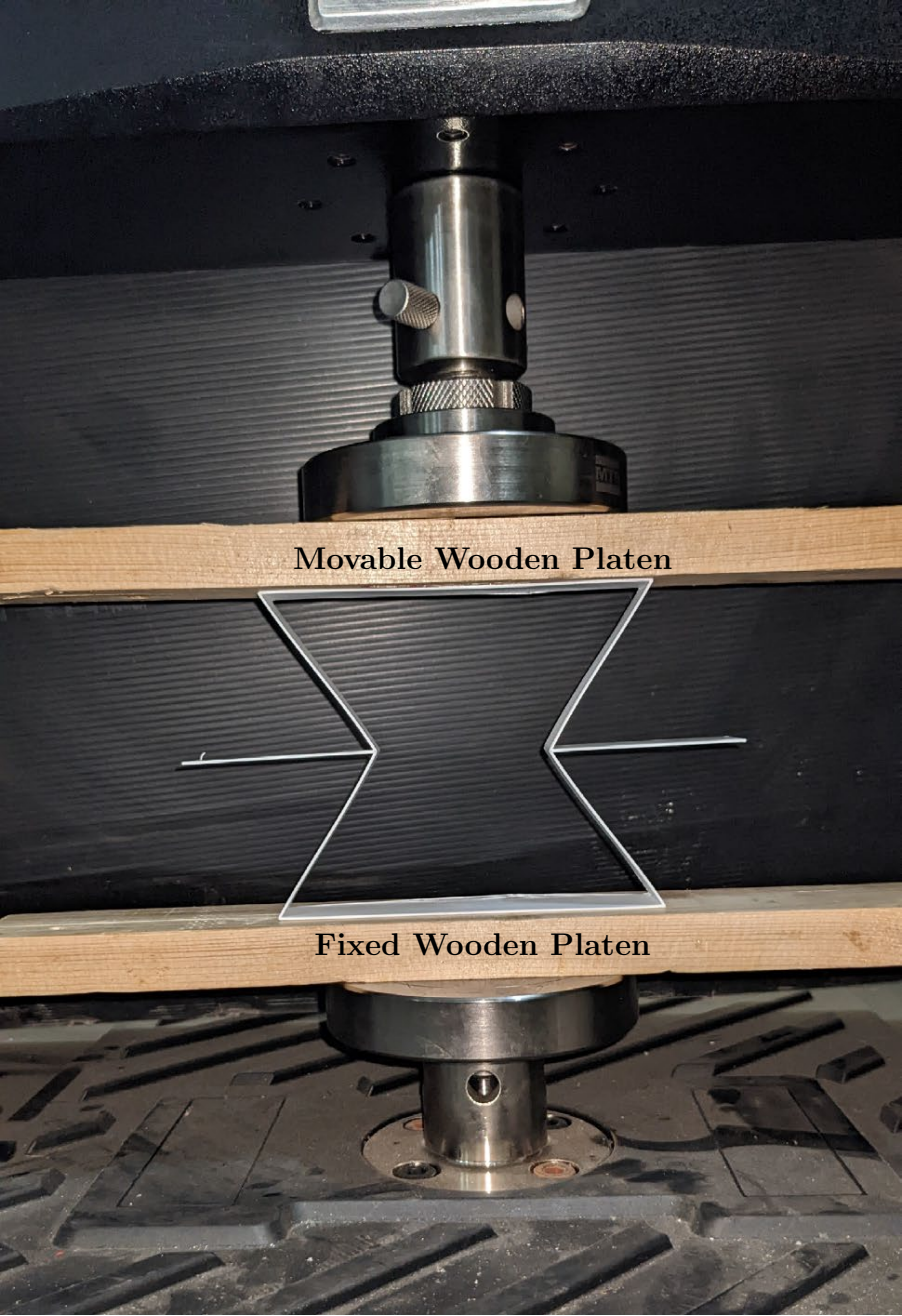
Test setup.

The mechanical properties of the auxetic unit cells under compression loading were tested using a 3kN load cell Instron UTM machine (Instron 6800 series). Figure 8 shows the experimental setup of a re-entrant sample. The tests were performed by placing the specimen on a fixed wooden platen attached to the circular platen of the testing machine. The displacement rate was set at a constant value of 0.0333 mm/sec to observe a quasi-steady mechanical response.

The deformation of specimens in all experimental tests was captured and recorded using a digital camera. The deformed specimen’s images (frames) were then captured at a rate of 30 frames per second. The instantaneous change in the axial and lateral displacements of the traced geometry was calculated using MATLAB. The average distance between the top and bottom plates was taken as the axial displacement. At the same time, the average displacement between the left and right structural columns was taken as the lateral displacement. The accurate axial and lateral deformation was calculated accordingly, in which at each image (i), the preceded image (i − 1) was used as a reference. Poisson’s ratio was calculated till the corner of the re-entrant unit cell came in contact with each other.

### Quasi-steady compression test response

**Figure 9:**
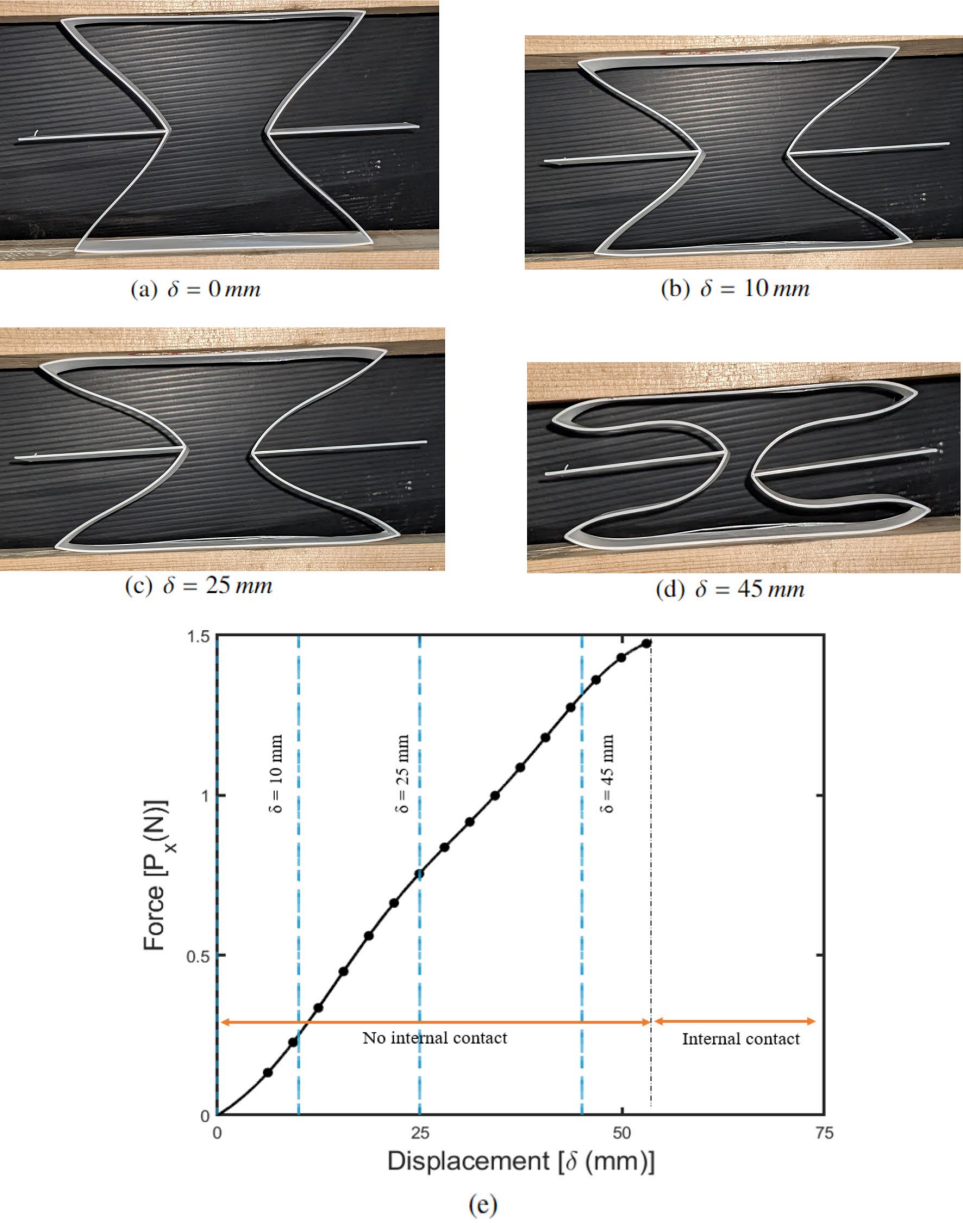
Results of the quasi-steady compression test of 2D re-entrant auxetic structure with photographs taken during the test at various moving head displacements[(**a**)–(**d**)], and (**e**) force–displacement response for the test configuration.

The experimental response for the 2D re-entrant auxetic structure is shown in Fig. 9. The response of the structure under compression load can be divided into two parts represented by (1) no internal contact, and (2) internal contact. The compression loading causes an inward contraction of the auxetic cell leading to a lateral deformation of inclined walls.

Due to the internal contraction of the cell, the wall corners come in contact with the horizontal wall leading to contact-related forces. The inclined walls are subjected to both bending moment and axial compression simultaneously leading to a buckled deformation response . The deformation response due to internal wall rotation and movement is not symmetric in nature, as shown in Fig. 9d. This can be caused due to the rotation of boundary corners during the compressive loading of the auxetic structure under UTM. For the purpose of comparison with the numerical results, the compression response of the specimen in the region with no internal contact was considered.

## Results and discussions

In this section, the effective elastic properties of the 2D and 3D re-entrant type geometries are presented as a function of applied loads. As emphasised in "Numerical methodology", the members constituting the auxetic microstructure are assumed to be thin strips and circular tubes. This allows the investigation of the influence of non-classical non-linearities. Simulations also reveal, quantitatively, the improvement achieved in auxetic behaviour for the geometries under large deformations.

For the 2D re-entrant geometry, the in-plane Poisson’s ratio, i.e., ${v}_{yx}$ is presented as a function of applied far-field horizontal load, i.e., ${P}_{x}$. Similarly, for the 3D re-entrant geometry, the variation of Poisson’s ratio along the vertical direction, i.e., ${v}_{xz}$ is shown w.r.t. the load along the vertical direction, i.e., ${P}_{z}$. The rib-inclination angle for the re-entrant type geometry of the micro-structure significantly affects the macro-scale behaviour. Hence, the variation of Poisson’s ratio with applied far-field stresses was determined for different values of rib-inclination angles for a thorough parametric evaluation. For the 2D as well as 3D re-entrant type geometry, the ratio of the length of the vertical members to the inclined members, i.e., $H/L$ was taken as 2, and the material for the auxetic frame was assumed to be ABS plastic, for which E = 2.2 GPa and ν = 0.37. The value of Poisson’s ratio under small deformation, as determined from Eq. (2) for the 2D re-entrant geometry and Eq. (4) for the 3D re-entrant geometry are summarized, for reference, in Tables 3, 4 and 5 for the geometric parameters considered in this study for numerical simulations.

**Table 3:** Elastic Properties for 2D re-entrant geometry constituted of thin strips.

Model parameters					Small Deformation Theory	GEBT		
*θ* (°)	*H* (mm)	*L* (mm)	*b* (mm)	*t* (mm)	${v}_{yx}$	${v}_{L}$	${v}_{NL}$	η (%)
20	160	80	10	0.2	−1.55	−0.244	−0.5	107
				0.25	−1.55	−0.249	−0.46	84
				0.3	−1.55	−0.279	−0.452	61
30	160	80	10	0.2	−1	−0.24	−0.423	76
				0.25	−1	−0.305	−0.437	43
				0.3	−1	−0.338	−0.439	29
40	160	80	10	0.2	−0.672	−0.306	−0.382	25
				0.25	−0.672	−0.333	−0.385	15
				0.3	−0.672	−0.351	−0.387	10
50	160	80	10	0.2	−0.437	−0.28	−0.298	6.5
				0.25	−0.437	−0.292	−0.303	3.7
				0.3	−0.437	−0.297	−0.305	2.5

**Table 4:** Elastic properties for 2D re-entrant geometry constituted of thin circular tubes.

Model parameters					Small Deformation Theory	GEBT		
*θ* (°)	*H* (mm)	*L* (mm)	*R* (mm)	*t* (mm)	${v}_{yx}$	${v}_{L}$	${v}_{NL}$	η (%)
30	160	80	5	0.2	−0.915	−1.18	−1.01	17
				0.3	−0.915	−1.17	−1.04	12
				0.4	−0.915	−1.12	−1.05	6
40	160	80	5	0.2	−0.637	−0.82	−0.70	16
				0.3	−0.636	−0.81	−0.73	11
				0.4	−0.638	−0.77	−0.74	5.2
50	160	80	5	0.2	−0.421	−0.57	−0.47	15.8
				0.3	−0.421	−0.54	−0.48	10.2
				0.4	−0.421	−0.54	−0.50	5
60	160	80	5	0.2	−0.246	−0.32	−0.28	14
				0.3	−0.247	−0.33	−0.29	7
				0.4	−0.247	−0.31	−0.297	3

**Table 5:** Elastic properties for 3D re-entrant geometry constituted of thin circular tubes.

Model parameters					Small Deformation Theory	GEBT		
*θ *(°)	*H* (mm)	*L* (mm)	*R* (mm)	*t* (mm)	${v}_{xz}$	${v}_{L}$	${v}_{NL}$	η (%)
30	160	80	5	0.2	−3.451	−3.72	−4.24	14
				0.3	−3.459	−3.79	−4.07	7.5
				0.4	−3.468	−3.81	−3.97	4.2
40	160	80	5	0.2	−2.111	−2.33	−2.82	21
				0.3	−2.119	−2.39	−2.70	12.6
				0.4	−2.117	−2.43	−2.59	6.6
50	160	80	5	0.2	−1.404	−1.57	−1.92	22
				0.3	−1.404	−1.61	−1.83	13.2
				0.4	−1.407	−1.64	−1.74	6
60	160	80	5	0.2	−0.955	−1.08	−1.35	24
				0.3	−0.956	−1.12	−1.28	13.6
				0.4	−0.957	−1.14	−1.22	6.4

For the 2D and the 3D geometries, as the applied loads gradually increase and the effect of non-linearity becomes more pronounced, the value of Poisson’s ratio deviates significantly from the values tabulated in Tables 3, 4 and 5. This deviation from the values for elastic constants as determined from the stress-independent analytical expressions is attributed to large deflections as well as the cross-sectional warping of the members constituting the auxetic frame. Specifically, to quantify the influence of non-classical non-linearity arising due to cross-sectional warping of the ligaments, on the effective Poisson’s ratio, the following parameter η is introduced:

12 $$\upeta =| \frac{{ v}_{NL}-{ v}_{L}}{{ v}_{L}}|\times 100\%$$

In Eq. (12), ${v}_{NL}$ is the Poisson’s ratio determined as per Eqs. (1) and (3) for the geometries by considering the variational asymptotic method-based formulations for the non-linear cross-sectional stiffness matrices presented in “Numerical methodology”. For both thin strip and thin circular tube cases considered, the cross-sectional matrices account for the nonlinear variations of bending and torsional stiffness of the members with 1-D strains along the beam reference line of the half-length cantilever (see Fig. 2 and 3). ${v}_{L}$ is the value of Poisson’s ratio determined considering cross-sections with equivalent bending stiffness (i.e., square for thin strips and circular for thin tubes).

In the following sections, the variation of the Poisson’s ratio w.r.t the applied loads has been presented for thin strips ("Strip-based structural member:") and circular tubes ("Tubular geometry"). The value of the quantifying parameters, i.e., η as determined for different geometric parameters has been indicated in the plots to highlight the observed improvement in the auxetic behaviour.

### Strip-based structural member

#### Numerical results

**Figure 10:**
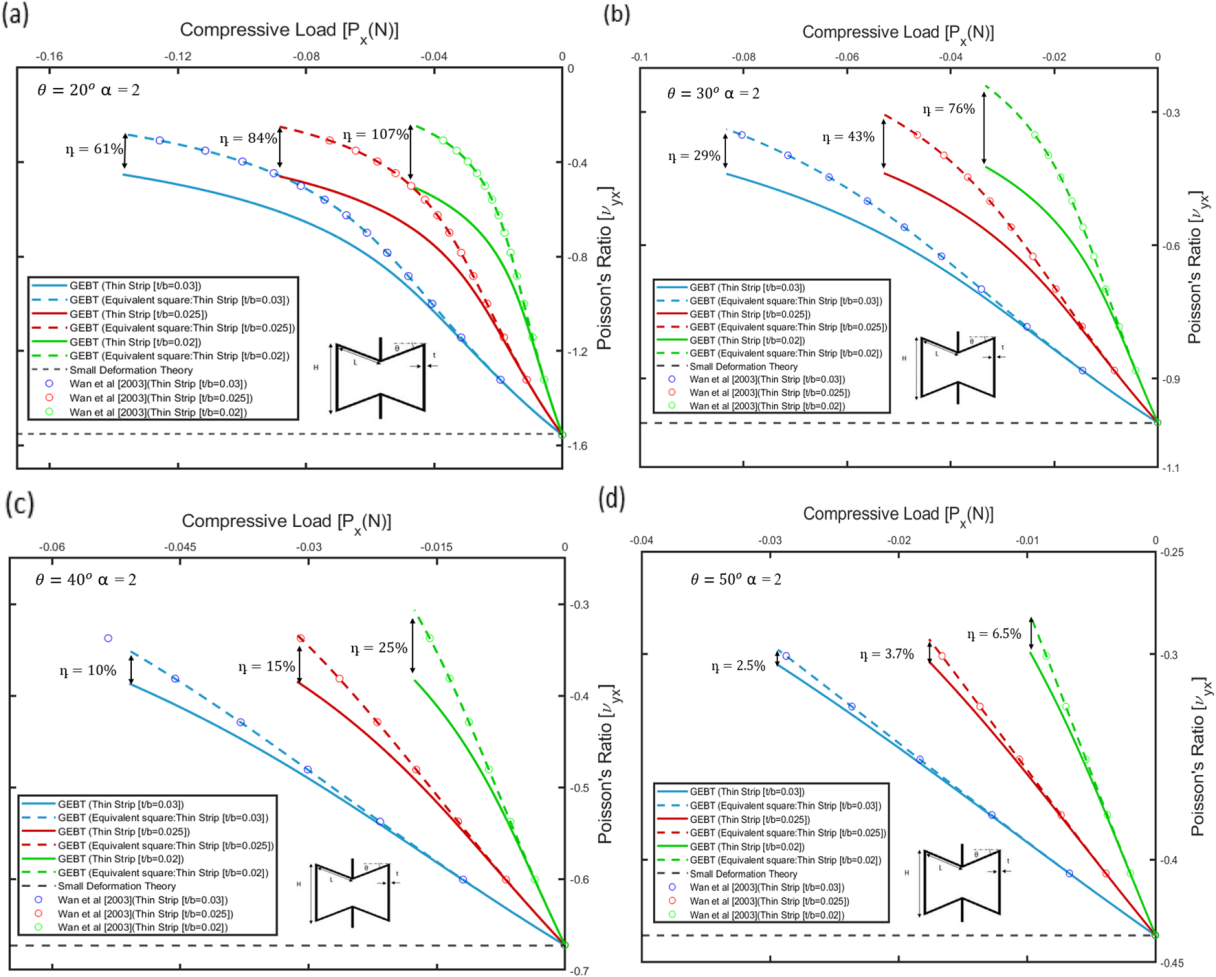
Variation of Poisson's ratio, i.e., ${v}_{yx}$ with applied far-field compressive load in the horizontal direction, i.e., ${P}_{x}$ for different rib inclination angles: (**a** $)\theta =2{0}^{\circ}$, (**b**) $\theta =3{0}^{\circ}$, (**c**) $\theta =4{0}^{\circ}$ and (**d**) $\theta =5{0}^{\circ}$. Variation of Poisson's ratio with applied far-field stress is determined for different values of thickness to width ratio of the strips, i.e., $[t/b = 0.02, 0.025, 0.03]$. The results are validated with plots for Poisson's ratio determined from the elastica model presented by Wan et al.^40^. The plots for linear cross-sectional stiffness matrix are shown by dashed lines and the plots for non-linear cross-sectional stiffness matrix are shown by solid lines.

The dimensions of the strip were taken such that the ratio of the thickness to the width of the strip, i.e., ${\delta }_{t}$ is sufficiently small. Hence, the influence of non-linearity on the auxetic behaviour of the re-entrant geometry is evaluated considering ${\delta }_{t}$ = 0.02, 0.025 and 0.03 associated with different rib inclination angles, i.e., $\theta =$ 20°, 30°, 40° and 50°. For the 2D re-entrant geometry, the variation of the Poisson’s ratio against the applied load, i.e., ${\nu}_{yx}$ vs. ${P}_{x}$ is plotted in Fig. 10a–d. It is significant to mention that under large loads the members of the auxetic frame would come into contact due to internal contraction of the cell as shown in the test response for the configurations in "Experimental methodology". Since the present work does not take into consideration the subsequent change in auxetic behaviour, the variation of Poisson’s ratio is plotted over the range of applied loads for which the internal collision between the members is not evident. The values for Poisson’s ratio, i.e., ${v}_{yx}$ for the 2D re-entrant geometry are plotted considering the members constituting the auxetic frame to have a thin rectangular cross-section (thin strips), as well as for members with a square cross-section with equivalent bending stiffness to the strips. Considering a linear cross-sectional stiffness matrix for the equivalent square cross-section as the geometry deforms under in-plane compression, Poisson’s ratio, i.e., ${v}_{L}$ varies significantly from the values predicted by small deformation theory, i.e., Eq. (2) and becomes increasingly less negative as shown in Fig. 10a–d, hence implying that the auxetic behaviour for the geometry undergoes progressive degradation under large deformation. For a square cross-section, since cross-sectional warping is not significant, the deviation from the values predicted by the load-independent analytical formulations for the Poisson’s ratio, i.e., values tabulated in Table 3, is primarily attributed only to one-dimensional nonlinearity along the beam reference line or large deflections of the members constituting the auxetic frame. This behaviour for the geometry predicted by the geometrically exact beam theory for equivalent square cross-section was compared with results from the elastica model presented by Wan et al.^40^ for the 2D re-entrant geometry for similar model parameters as shown in Fig. 10a–d. Since, the elastica model, similar to the assumptions of Euler–Bernoulli beam theory neglects cross-sectional warping, the values agree well.

For thin strips accounting for the cross-sectional warping, i.e., considering the non-linear cross sectional stiffness matrix, wherein the terms of the stiffness matrix vary with one-dimensional strains along the beam reference line, in Fig. 10a–d it is observed that over the same range of applied loads, the value of Poisson’s ratio, i.e., ${v}_{NL}$ is more negative compared to ${v}_{L}$. This implies that the auxetic behaviour improves significantly, which is also captured by the value of the parameter η, which increases up to 107%, for t/b = 0.02 and rib-inclination angle, i.e., $\theta =$ 20° (ref. Table 3). This implies that the non-classical non-linear effect becomes significant and enhances the auxetic behaviour.

Further from Fig. 10a–d, it can be inferred that the value of the quantifying parameter η is higher for lower values of ${\delta }_{t}$ i.e., t/b. Specifically, the non-linear effect is attributed to the increasing magnitude of the bending stiffness corresponding to curvature ${\kappa }_{2}({\kappa}_{1}=0,{\kappa}_{3}=0)$ (ref. “Strip-based structural member”):

13 $$[{S}_{22}{]}_{NL}=\frac{Eb{t}^{3}}{12}\left[1+\frac{{b}^{2}{v}^{2}}{60(t/b{)}^{2}}{\kappa }_{2}^{2}\right]$$

Equation (13) for the bending stiffness can be reframed as follows:

14 $$\frac{ [{S}_{22}{]}_{NL}}{[{S}_{22}{]}_{L}}=\left[1+\frac{{b}^{2}{v}^{2}}{60(t/b{)}^{2}}{\kappa }_{2}^{2}\right]$$

**Figure 11:**
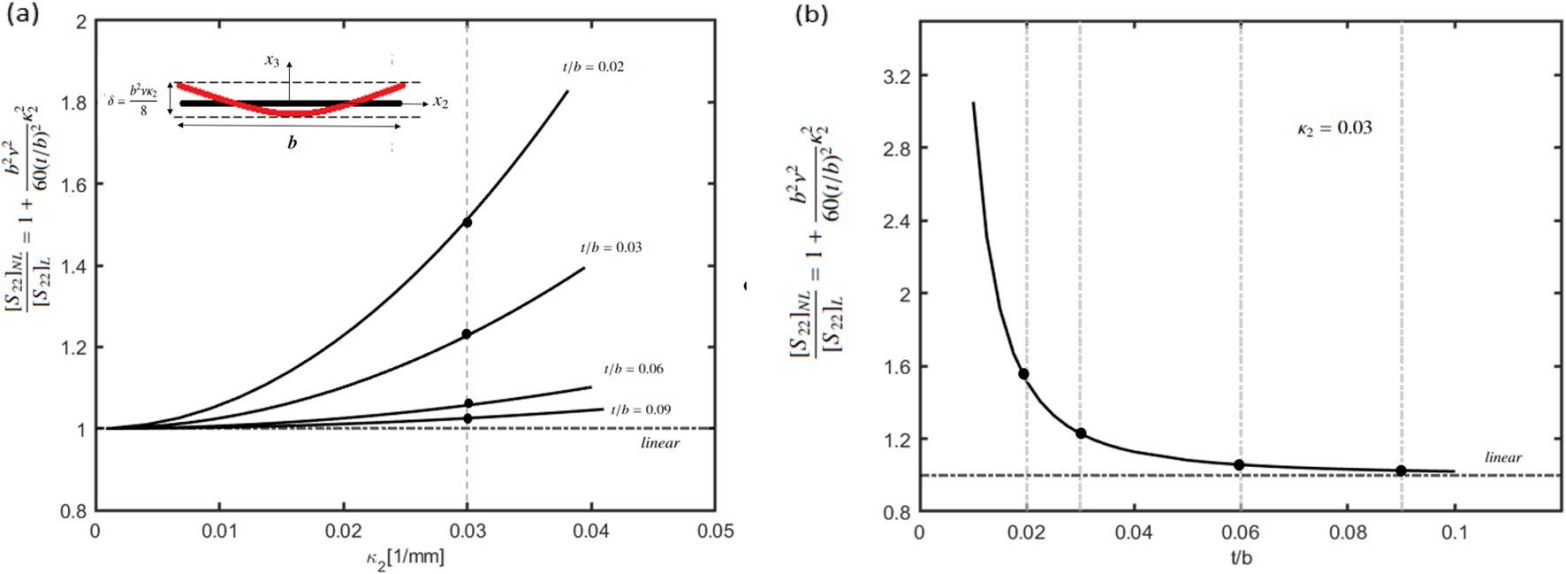
(**a**) Variation of relative increase in the value of bending stiffness, i.e., $\frac{{[S}_{22}{]}_{NL}}{[{S}_{22}{]}_{L}}$ with the curvature, i.e., ${\kappa }_{2}$ and shape of the deformed cross-section for thin strip (b = 10 mm, v = 0.37 ref. Eq. 6). (**b**) Variation of relative increase in the value of bending stiffness with ${\delta }_{t}$. Increase in the cross-sectional stiffness term corresponding to ${\kappa }_{2}$ due to non linearity is inversely related to ${\delta }_{t}$.

It can be inferred from Eq. (14), that relative increase in the cross-sectional stiffness term corresponding to ${\kappa }_{2}$ due to non-linearity, i.e., $\frac{{[S}_{22}{]}_{NL}}{[{S}_{22}{]}_{L}}$ is inversely related to ${\delta }_{t}$ . Figure 11a shows the variation of relative increase in the value of bending stiffness, i.e., $\frac{{[S}_{22}{]}_{NL}}{[{S}_{22}{]}_{L}}$ with the curvature, i.e., ${\kappa }_{2}$ and Fig. 11b shows the variation of the ratio with ${\delta }_{t}$. From Fig. 11 it can be inferred that for higher values of ${\delta }_{t}$ the ratio converges to 1, i.e., the linear theory, wherein the bending stiffness is assumed to be constant and only large deformations of the inclined members influences the load dependent behaviour for the geometry, similar to the elastica model given by Wan et al.^40^. Figure 11a also shows the shape of the warped cross-section (ref. Eq. 6), wherein δ is the relative deformation between the central (i.e., ${x}_{2}$ = 0) and extreme positions (i.e., ${x}_{2}$= b/2) along the width of the cross-section (ref Fig. 4).

**Figure 12:**
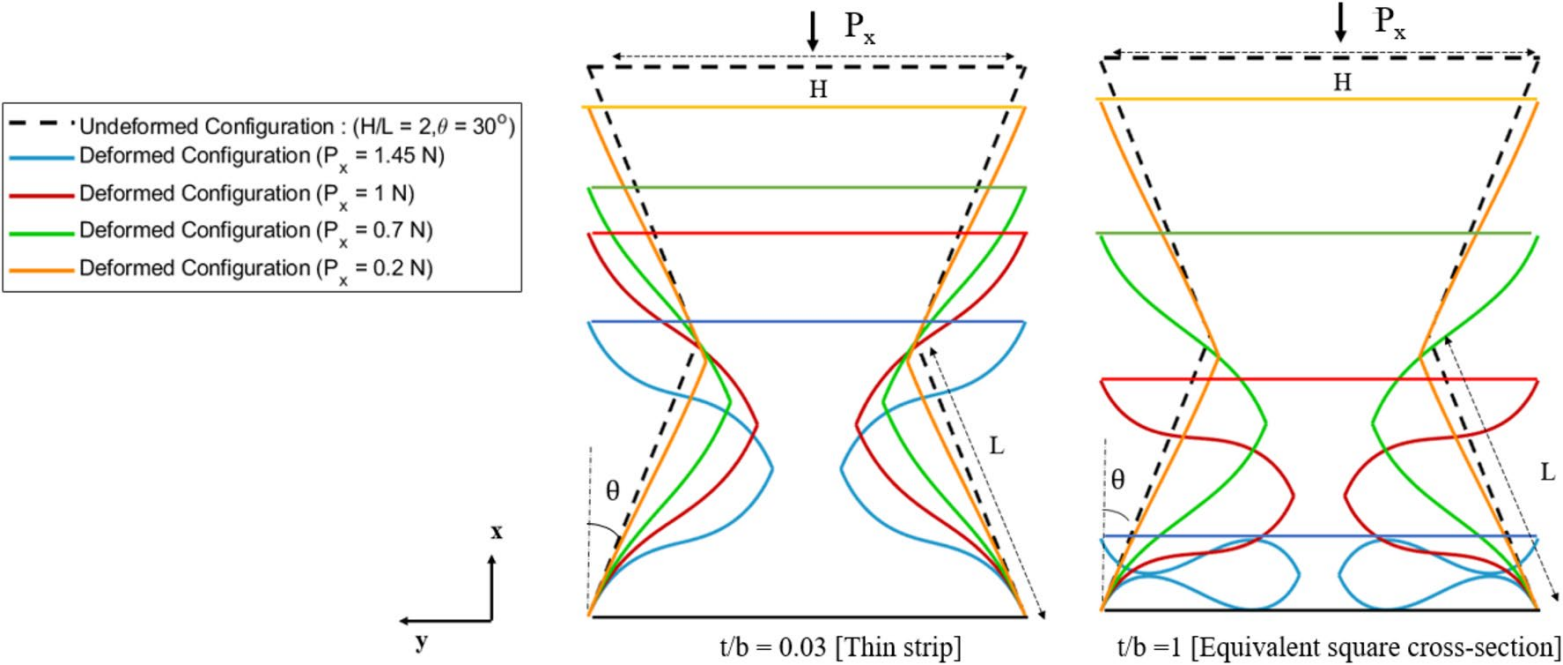
Deformed configuration for 2D re-entrant geometry (H/L = 2, $\theta =3{0}^{\circ}$,L = 8 cm,${\delta }_{t}$ = 0.03) at different magnitudes of applied loads (${P}_{x}$ = 1.45,1,0.7 and 0.2 N) for thin strip and equivalent square cross-section. The increase in the cross-sectional stiffness delays internal contact between the cell walls of the unit cell.

As a consequence of increasing cross-sectional stiffness the deformation of the inclined member is reduced, thereby increasing the range of load over which auxetic behaviour is retained. Figure 12 also shows the deformed configuration for the tested specimen in "Experimental methodology", from the numerical simulations at different magnitudes of applied loads (${P}_{x}$ = 1.45, 1, 0.7 and 0.2 N). On comparing the deformed configuration for thin strips and for equivalent square cross-section, it is inferred that the deformation of the inclined members is significantly reduced by the non-linear effect when compared with the geometry constituted of members with equivalent square cross-sections. This phenomenon also delays the onset of internal contact between the cell members, thereby increasing the range of applied load over which the geometry exhibits auxetic behaviour. Additionally, it is also observed that the effect of non-linearity, and thereby the associated improvement in auxetic behaviour is relatively more pronounced for lower values of the rib-inclination angles. For higher values of rib-inclination angles, the internal collision of the members initiates at relatively lower values of the loads due to reduced spacing between the cell walls, thereby limiting the effect of non-linearity arising due to cross-sectional warping.

It is also significant to mention here that the analysis in this work is limited to a single unit cell and with reference to the half-length cantilever beam model for the re-entrant geometry given by Wan et al.^ 40^, the joint rotations are suppressed in the proposed framework, to impose periodic boundary conditions on the geometry, in order to represent a continuum environment. Although the non-linear effect is governed by cross-sectional warping of the thin strip, warping itself is symmetric (ref. Fig. 11a), affected by in-plane bending of the inclined members, i.e., ${\kappa }_{2}$ and is independent of the boundary conditions at the joints as can be inferred from Eq. (6). Hence, although the numerical model in the present work, is limited to a single unit cell, it is expected that the non-linear effect would be significant for a larger representative volume as well.

Traditionally, re-entrant honeycombs are constructed with filleted corners to minimize localised stress concentrations. The current formulation does not directly account for added stiffness of the connected region due to the fillet, however the framework can be extended to capture variation of cross-sectional stiffness along the length of the inclined members due to the added material at joints. In such a scenario, added material at the joint coupled with non-linearity exhibited by thin strips would potentially improve auxetic behaviour further.

#### Experimental verification

**Figure 13:**
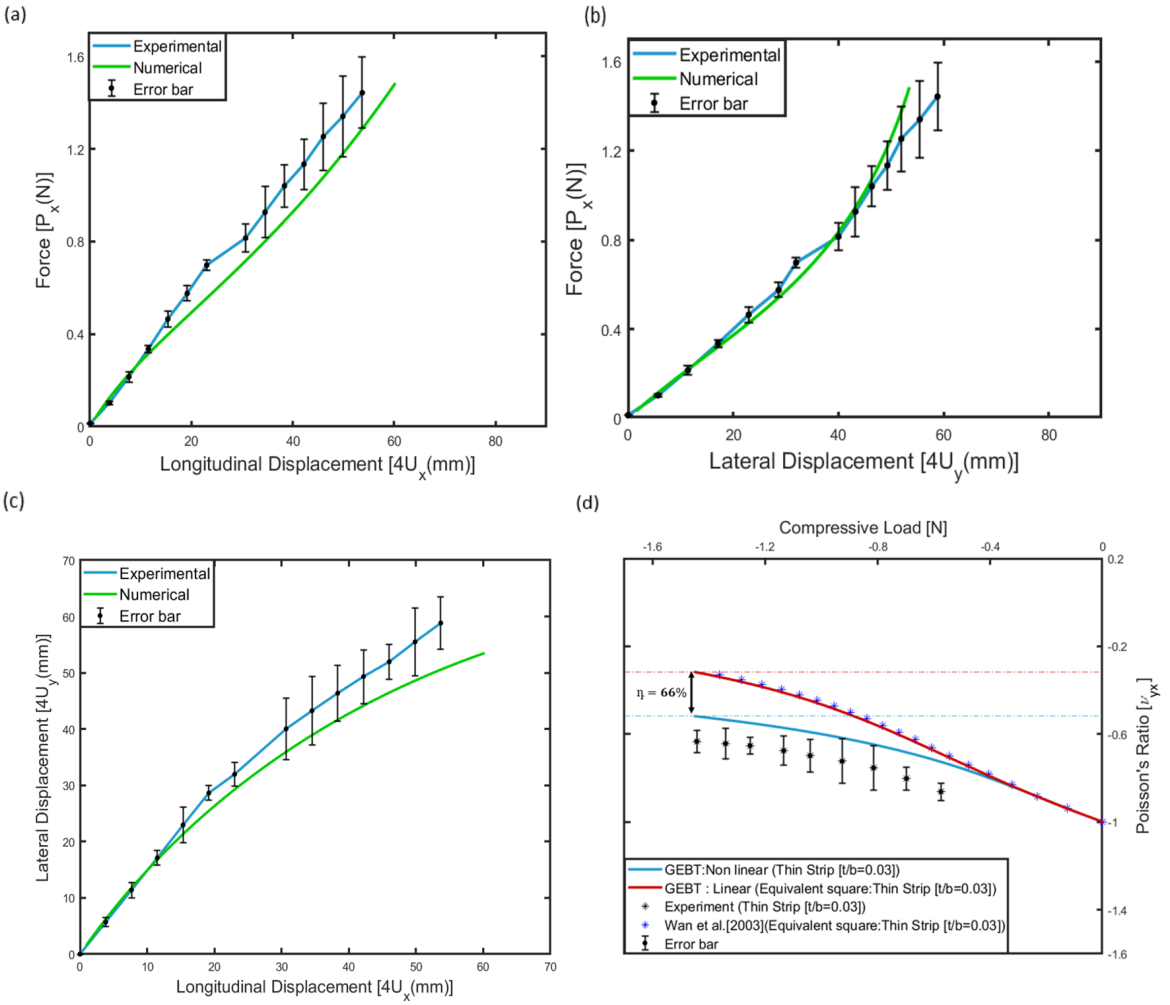
Comparison of numerical and experimental results for 2D re-entrant geometry : (**a**) load vs longitudinal displacement ($${P}_{x}$$ vs $${4U}_{x}$$). (**b**) Load vs lateral displacement ($${P}_{x}$$ vs $${4U}_{y}$$). (**c**) Lateral vs longitudinal displacement ($${4U}_{y}$$ vs $${4U}_{x}$$). (**d**) Variation of Poisson's ratio, i.e. $${v}_{yx}$$ with applied far-field compressive load in the horizontal direction, i.e. $${P}_{x}$$ for the test specimen (ref. Table 2: $$, \theta = 3{0}^{\circ}$$, $$, H/L = 2)$$.

The behaviour of the 2D re-entrant geometry constituted of thin strips was validated with the experimental results as shown in Fig. 13. The dimensions of the test specimen are tabulated in Table 2. The experimental validation for the numerical methodology and the associated improvement in auxetic behaviour is limited to the range of loads for which the internal contact between the members of the unit cell is not observed, i.e., the no contact region of the plot in Fig. 9. Figure 13a, b show the comparison between the load vs longitudinal (x-direction) and lateral (y-direction) displacement plots as predicted by the numerical methodology (Fig. 6) and the experimental results ("Experimental methodology"). Figure 13c also shows the lateral displacement plotted against the longitudinal displacement. The experimental and numerical values of displacements are in reasonable agreement with an upper bound of 20% on the deviation. Further, Fig. 13d shows the comparison between the load vs Poisson’s ratio behaviour. The numerical results are also plotted for the 2D re-entrant geometry constituted of members with a square cross-section of equivalent bending stiffness to highlight the improvement in auxetic behaviour. From the comparison, it is inferred that the numerical results for the thin strip agree reasonably well with the experimental response, and the non-linearity arising due to cross-sectional warping for the thin strip improves auxetic behaviour significantly. With reference to Eq. (6), the shape of the warped cross-section is parabolic, however the maximum value of δ < 1 mm (ref. Fig. 11a) for the tested geometry, and hence could not be measured due to the limitations of the available experimental setup, however, the effect of increased bending stiffness due to the deformed shape of the cross-section is captured by the significant reduction in the deformations when compared with the results from linear theory, wherein, the terms of the stiffness matrix are assumed to be curvature independent constants. It is also highlighted here, that the warping is within the plane of the geometry, and out of the plane of the thin strip. The numerical plot indicates that the value of the quantifying parameter η increases up to 66% for the tested configuration (ref. Table 6). The experimental results for the tested configuration also capture the non-linear behaviour, and align well with the numerical predictions. Figure 13d also shows the error bar on the measured value of Poisson’s ratio. The deviation between numerical and experimental results is attributed to the difference in the applied boundary conditions on the numerical model and the tested specimen, i.e., the boundary conditions are different for the present experimental set-up, in the sense that it is not periodic but resembling a displacement-driven hard boundary conditions with one end fixed and the other end being displaced (Fig. 8). Hence, comparison of the experimental results with the numerical results is not one-to-one. However, the results of the numerical work would resemble the larger RVE system due to the periodic boundary conditions applied to the model in the current work.

**Table 6:** Comparison of numerical and experimental results for 2D strip-based geometry.

Model parameters					Linear	Load	GEBT			Experiment	
	*H* (mm)	*L* (mm)	*b* (mm)	*t* (mm)	${v}_{yx}$	${P}_{x}(N)$	${v}_{L}$	${v}_{NL}$	*n* %	${v}_{exp}$	%Error
30	160	80	20	0.6	-1	1.485	-0.31	-0.52	66	-0.63	21

### Tubular geometry

**Figure 14:**
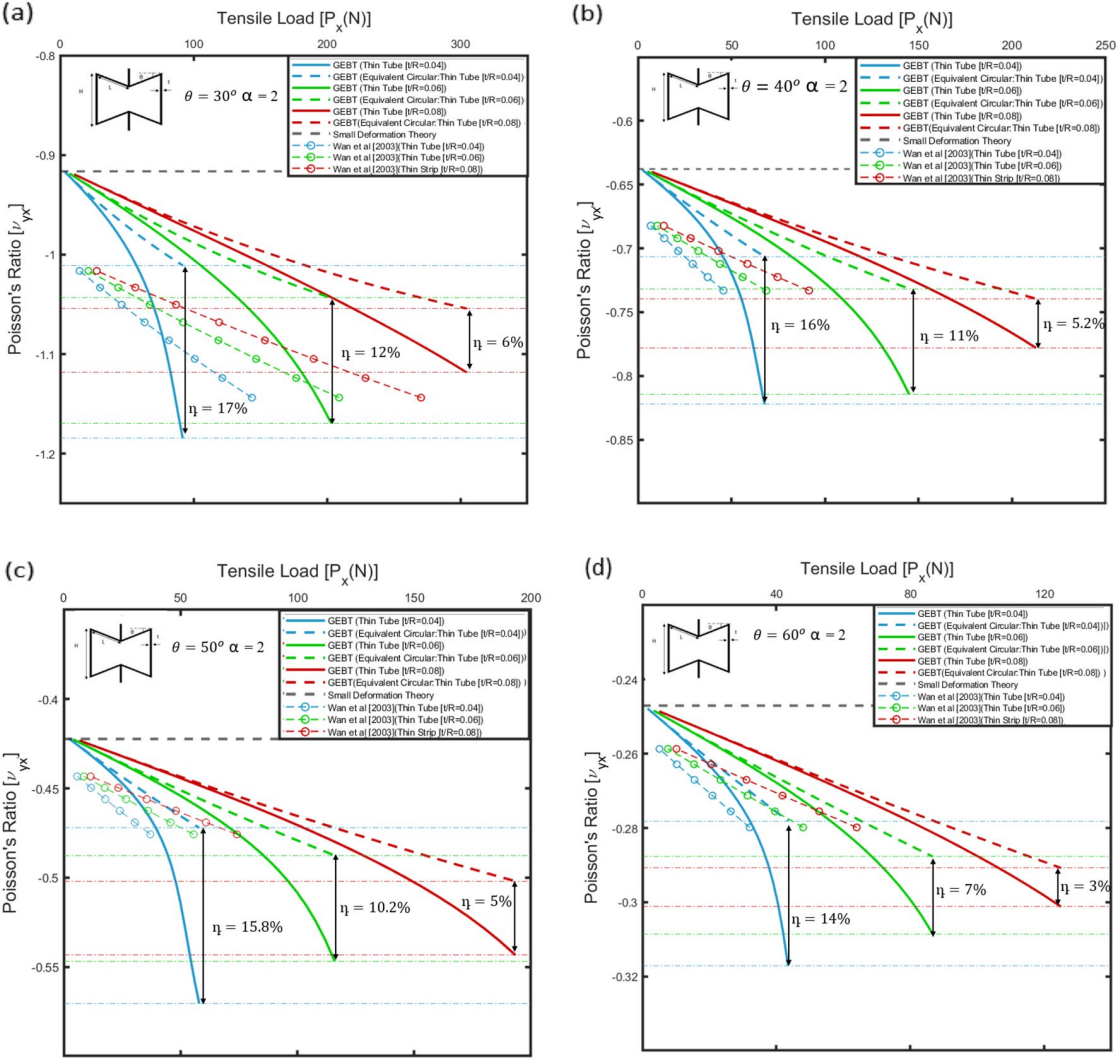
Variation of Poisson's ratio, i.e., ${v}_{yx}$ with applied load in the horizontal direction, i.e., ${P}_{x}$ for different rib inclination angles: (**a**) $\theta =3{0}^{\circ}$, (**b**) $\theta =4{0}^{\circ},$ (**c**) $\theta =5{0}^{\circ}$ and (**d**) $\theta =6{0}^{\circ}$. Variation of Poisson's ratio with applied far-field stress is determined for different values of thickness to the radius of the circular tubes, i.e., $[t/R = 0.04, 0.06, 0.08]$. The results are validated with plots for Poisson's ratio determined from the elastica model presented by Wan et al.^40^. The plots for linear cross-sectional stiffness matrix are shown by dashed lines and the plots for non-linear cross-sectional stiffness matrix are shown by solid lines.

**Figure 15:**
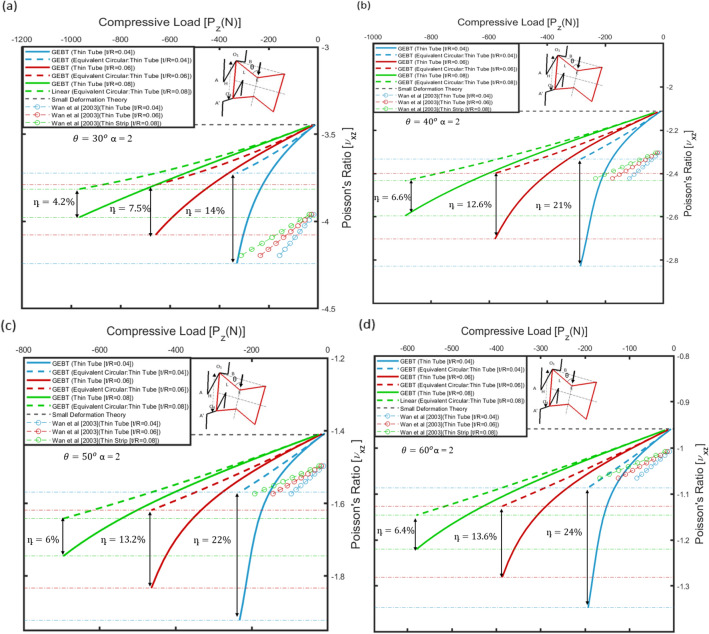
Variation of Poisson's ratio, i.e., ${v}_{xz}$ with applied load in the vertical direction, i.e., ${P}_{z}$ for different rib inclination angles: (**a**) $\theta =3{0}^{\circ}$, (**b**) $\theta =4{0}^{\circ},$ (**c**) $\theta =5{0}^{\circ}$ and (**d**) $\theta =6{0}^{\circ}$. Variation of Poisson's ratio with applied far-field stress is determined for different values of thickness to the radius of the circular tubes, i.e., $[t/R = 0.04, 0.06, 0.08]$. The results are validated with plots for Poisson's ratio determined from the elastica model presented by Yang et al.^49^. The plots for linear cross-sectional stiffness matrix are shown by dashed lines and the plots for non-linear cross-sectional stiffness matrix are shown by solid lines.

The dimensions of the circular tube were taken such that the ratio of the thickness to the radius of the circular tube i.e., the parameter ${\delta }_{h}$ is sufficiently small. The influence of the ovalisation induced non-linearity on the auxetic behaviour is investigated considering ${\delta }_{h}=0.04, 0.06$ and 0.08 in association with the rib-inclination angles $\theta =$ 30°, 40°, 50° and 60° for the 2D re-entrant geometry and 3D re-entrant geometry. Figure 14a–d show the variation of the Poisson’s ratio, i.e., ${v}_{yx}$ with applied far-field tensile load, i.e., ${P}_{x}$ for the 2D re-entrant geometry and Fig. 15a–d show the variation of the Poisson’s ratio ${v}_{xz}$ with applied far-field compressive load, i.e., ${P}_{z}$ for the 3D re-entrant geometry. The variation of Poisson’s ratio, i.e., ${v}_{yx}$ and ${v}_{xz}$ is plotted considering the members constituting the auxetic frame to have a thin tubular cross-section, as well as for members with a circular cross-section with equivalent bending stiffness to the thin tubes. For the circular cross-section with equivalent bending stiffness, the cross-sectional warping is negligible, thereby implying that the bending stiffness (${S}_{22}$/${S}_{33}$ ref. "Numerical methodology") would not vary with curvature. In this case, the Poisson’s ratio, i.e., ${v}_{L}$ becomes increasingly more negative under tensile loads in the horizontal direction, i.e., ${P}_{x}$ as shown in Fig. 14a–d for the 2D re-entrant geometry and under compressive loads in the vertical direction, i.e., ${P}_{z}$ for 3D re-entrant geometry as shown in Fig. 15a–d. The results indicate an improvement in auxetic behaviour for both geometries under the considered loading conditions.

As discussed in "Strip-based structural member:", for the equivalent circular cross-section, the deviation from the values predicted by small deformation theory (Tables 4 and 5) is attributed only to the one-dimensional non-linearity along the beam reference line of the inclined members. This behaviour predicted by the geometrically exact beam theory for constant cross-sectional stiffness of the members is validated with the results from the elastica model for similar geometric parameters^40,41^. The deviation in the values predicted by the elastica model and the geometrically exact beam theory is within an upper bound of 10%, and is attributed to the axial deformation of the half-length cantilever beam in Figs. 2 and 3, which is neglected in the formulations of the elastica theory.

**Figure 16:**
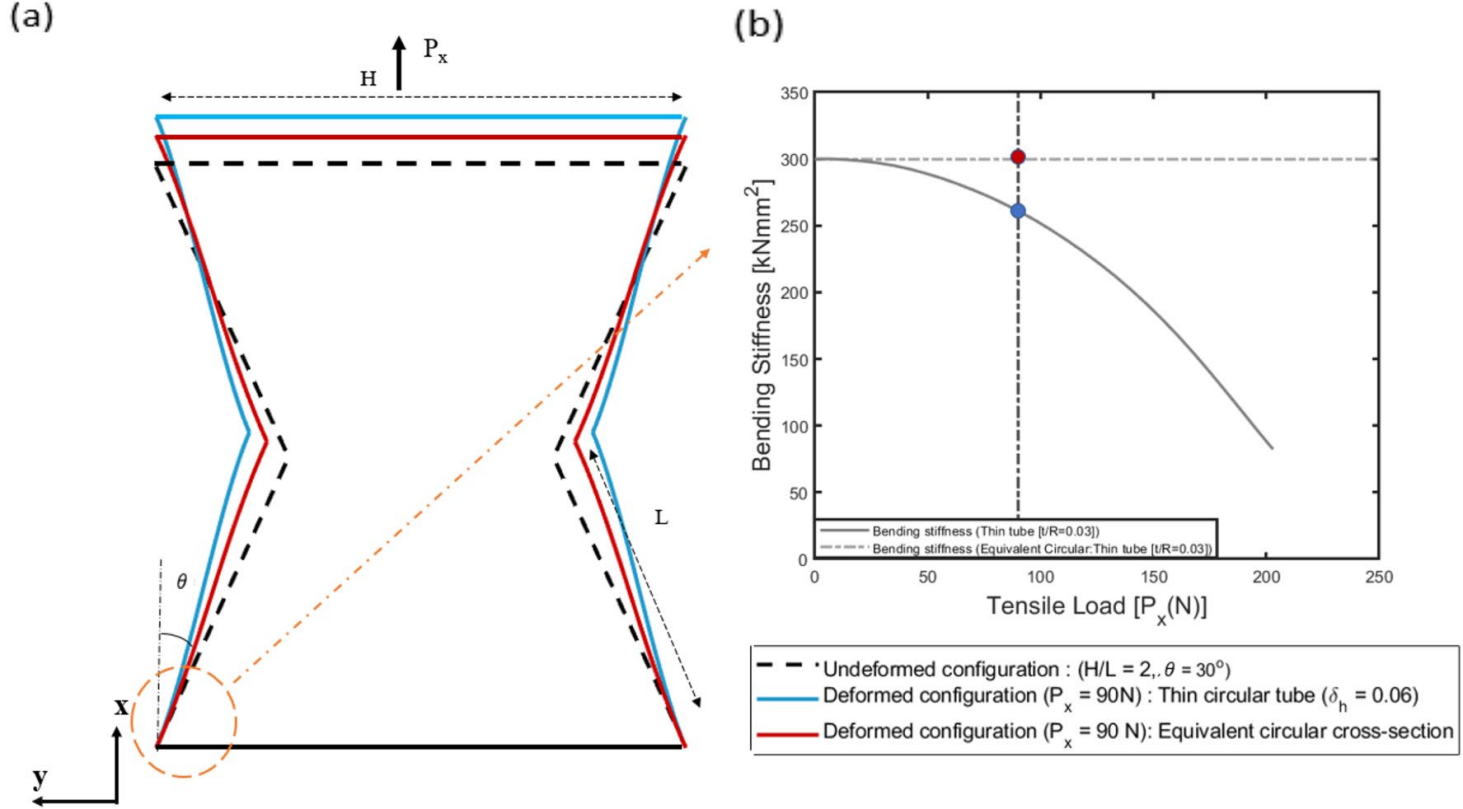
(**a**) Deformed configuration for 2D re-entrant geometry (H/L = 2, $, \theta = 3{0}^{\circ}$, L = 8 cm and $, {\delta }_{h} = 0.06$) under tensile applied load (${P}_{x}$= 90 N) for thin tube and equivalent circular cross-section and (**b**) cross-sectional bending stiffness $(kNm{m}^{2})$ plotted as a function of the applied load ${P}_{x}$.

For thin tubular cross-section, considering the non-linear cross-sectional stiffness matrix presented in "Numerical methodology", the value of Poisson’s ratio, i.e., ${v}_{NL}$ was observed to become progressively more negative compared to ${v}_{L}$ over the same range of applied tensile load for different rib-inclination angles. This, in turn, implies that the auxetic behaviour improves as the non-classical non-linear effect, specifically the Brazier’s effect becomes significant. The improvement in auxetic behaviour here is attributed to the decreasing bending stiffness as the curvature increases. This phenomenon is shown in Fig. 16b, wherein the cross-sectional bending stiffness for a section at the root of the half-length cantilever beam (H/L = 2, $\theta =3{0}^{0}$ , L = 8 cm, ${\delta }_{h}$ = 0.06) is plotted w.r.t. the applied tensile load, i.e., ${P}_{x}$. From Fig. 16b it is inferred that there is a significant reduction in the bending stiffness of thin tubes as compared to an equivalent circular cross-section. The effect of decreasing bending stiffness on the behaviour of the geometry is also captured by the deformed configuration of the geometry.

On comparing the deformed configuration of the geometry for thin tubes and for circular cross-sections with equivalent bending stiffness, it is inferred that as a consequence of the progressive reduction in bending stiffness with curvature, the thin tubes exhibit increased deformation, thereby resulting in higher negative values of Poisson’s ratio. The improvement in the auxetic behaviour is captured by the increasing values of the parameter η, which increases up to 17% for t/R = 0.04 and $\theta =3{0}^{0}$ for the 2D re-entrant geometry. From the plots, it is observed that the effect of non-linearity is relatively more pronounced for lower values of the parameter t/R. Further for the 3D re-entrant geometry, considering the linear cross-sectional stiffness matrix, the value of Poisson’s ratio becomes more negative under compressive far-field stress in the vertical direction, i.e., ${P}_{z}$ as shown in Fig. 15a–d. As in the case of the 2D re-entrant geometry, the deviation from the load -independent values from the small deformation theory for circular cross-section is attributed to one-dimensional non-linearity along the beam reference line of the inclined members. This behaviour for the 3D geometry under compression is validated with results from the elastica theory^49^. Accounting for the non-linear effects due to cross-sectional warping, the value of Poisson’s ratio ${v}_{NL}$ was observed to be more negative compared to ${v}_{L}$ over the same range of applied compressive load, thereby indicating that the auxetic behaviour improves as a consequence of non-linearity. For the 3D re-entrant geometry, the value of the parameter was observed to increase to 24% for $\theta =3{0}^{0}$ and t/R = 0.04.

## Conclusions

In this article, non-classical non-linear effects have been exploited through simple geometric engineering to improve the auxetic behaviour for the 2D and 3D re-entrant geometries under large deformations. The variational asymptotic two-dimensional cross-sectional analysis and geometrically exact beam theory were employed to determine the influence of such non-classical non-linearities on the variation of Poisson’s ratio with applied far-field stresses for different values of rib-inclination angles. Parametric evaluation dealing with the effect of rib-inclination angles, the ratio of the thickness to width for the strips and thickness to radius for the circular tubes on the auxeticity of the geometry has been presented.

- The numerical results suggest that the auxetic behaviour of the 2D re-entrant geometry with thin strips constituting the micro-structure, exhibited an improvement under compressive stress. This phenomenon is attributed to a progressive increase in stiffness constants corresponding to curvature due to which, the deformation of the inclined members undergoes significant reduction, allowing the geometry to retain auxetic behaviour over a larger range of applied stress. The numerical results indicated an improvement of up to 107% in the value of Poisson’s ratio.
- For the 2D re-entrant geometry under tensile stress, Poisson’s ratio becomes more negative for the unit cell constituted of thin circular tubes as the Brazier’s effect becomes significant, i.e., as the bending stiffness decreases with increasing curvature, enhancing the auxetic behaviour by up to 17%.
- For the 3D re-entrant geometry under far-field stresses in the z-direction, Brazier’s effect significantly improved the auxetic behaviour under compression, by up to 24%

With such simple geometric feature engineering at the member level of an auxetic microstructure, the results have shown a potential path to achieve enhanced auxetic behaviour across the existing range of auxetic configurations. It is also important to highlight that while exploiting such nonlinearities significantly increased the negative value of Poisson’s ratio, the overall stiffness decreases due to the very nature of the thin-walled members of the auxetic geometries considered.

### Supplementary Information

Supplementary Information.

## Data Availability

The datasets used and/or analysed during the current study are available from the corresponding author on reasonable request.
