# Supplementary material for: Exploiting nonlinearities through geometric engineering to enhance the auxetic behaviour in re-entrant honeycomb metamaterials
Source: Sci Rep. 2023 Nov 27;13:20915. doi: 10.1038/s41598-023-47525-7 (PMC10684525; doi:10.1038/s41598-023-47525-7)
Supplement: Supplementary file 1 — Supplementary Information. [file 41598_2023_47525_MOESM1_ESM.pdf]

Appendix: Exploiting nonlinearities through geometric engineering to enhance the auxetic behaviour in re-entrant honeycomb metamaterials

A. Strip-based structural member

In this section the mathematical formulations for determining the one-dimensional strain energy functional, i.e. U_{1D} in terms of one-dimensional strain measures along the beam reference line, i.e. γ_{11}, κ_1, κ_2 and κ_3 have been presented[43].

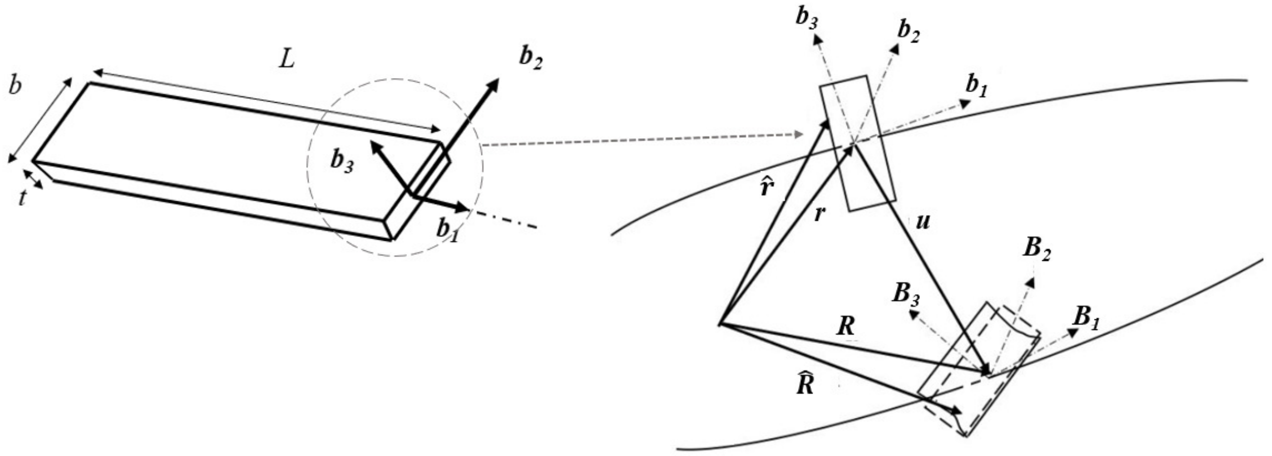

Figure A1: Thin strip with geometric parameters: length of member L, width b and thickness t: Undeformed and deformed configurations of the cross-section, position vector to a material point on the undeformed cross-section, i.e. $\hat{\boldsymbol{r}}$ is expressed in terms of measure numbers along the orthonormal triad of $\boldsymbol{b_i}$ vectors, and the position vector to a material point on the deformed cross-section, i.e. $\hat{\boldsymbol{R}}$ is expressed in terms of measure numbers along the orthonormal triad of $\boldsymbol{B_i}$ vectors.

From Fig. A1 it can be observed that $\boldsymbol{b_1}$ is directed along the beam reference line, which passes through the centroid of the cross-section while $\boldsymbol{b_2}$ and $\boldsymbol{b_3}$ are along two mutually perpendicular direction transverse to $\boldsymbol{b_1}$. These vectors form a basis such that the position vector, i.e. $\hat{\boldsymbol{r}}$ for any point on the cross-section of the beam is expressed in terms of the measure numbers along $\boldsymbol{b_1}$, $\boldsymbol{b_2}$ and $\boldsymbol{b_3}$, i.e.:

$$\hat{\boldsymbol{r}}(x_1, x_2, x_3) = x_1\boldsymbol{b_1} + x_2\boldsymbol{b_2}(x_1) + x_3\boldsymbol{b_3}(x_1) \tag{A.1}$$

As a consequence of the applied loads, each material point on the cross-section will undergo a rigid body translation and rotation, followed by warping. Hence, the position vector of a material

point on the deformed cross-section, i.e. $\hat{\boldsymbol{R}}$ is expressed as follows:

$$\hat{\boldsymbol{R}} = \boldsymbol{r} + \boldsymbol{u} + x_2\boldsymbol{B_2} + x_3\boldsymbol{B_3} + w_i\boldsymbol{B_i} \tag{A.2}$$

where $\boldsymbol{u}$ is the rigid body translation of the reference point, i.e. point of intersection of the reference line and the cross-section, $\boldsymbol{B_1}$ is normal to the deformed beam cross-sectional plane, while $\boldsymbol{B_2}$ and $\boldsymbol{B_3}$ are mutually perpendicular, lying in the plane of the deformed cross-section and $\boldsymbol{w}$ is the warping field. From the definition of covariant and contravariant vectors, the deformation gradient tensor $\underline{\boldsymbol{X}}$ is given by:

$$\underline{\boldsymbol{X}} = \boldsymbol{G_i}\boldsymbol{g^i} \tag{A.3}$$

where,

$$\boldsymbol{g^i} = \frac{\epsilon_{ijk}}{2\sqrt{g}}\boldsymbol{g_j} \times \boldsymbol{g_k} \tag{A.4}$$

$$\boldsymbol{g_1} = \frac{\partial \hat{\boldsymbol{r}}}{\partial x_1} \quad \boldsymbol{g_2} = \frac{\partial \hat{\boldsymbol{r}}}{\partial x_2} \quad \boldsymbol{g_3} = \frac{\partial \hat{\boldsymbol{r}}}{\partial x_3} \tag{A.5}$$

$$\boldsymbol{G_1} = \frac{\partial \hat{\boldsymbol{R}}}{\partial x_1} \quad \boldsymbol{G_2} = \frac{\partial \hat{\boldsymbol{R}}}{\partial x_2} \quad \boldsymbol{G_3} = \frac{\partial \hat{\boldsymbol{R}}}{\partial x_3} \tag{A.6}$$

The three-dimensional strains (Γ), are thereafter determined using the following relation for moderate local rotations[47]:

$$\Gamma = E - \frac{\tilde{X}.\tilde{X}}{2} + \frac{E\tilde{X} - \tilde{X}E}{2}$$
$$\tilde{X} = \frac{X - X^T}{2} - I$$
$$E = \frac{X + X^T}{2} - I$$

Further, the three-dimensional strains $\Gamma_{\alpha\beta}$, (α, β : 1, 2) are expressed in terms of the two - dimensional strains and curvatures, i.e. $\epsilon_{\alpha\beta}$ and $\rho_{\alpha\beta}$ along the mid-plane of the strips[43]:

$$\Gamma_{\alpha\beta} = \epsilon_{\alpha\beta} + x_3\rho_{\alpha\beta} \tag{A.7}$$

The strain energy functional can be expressed in terms of the membrane strains and curvatures i.e., $\epsilon_{\alpha\beta}$ and $\rho_{\alpha\beta}$, as well as the membrane extensional, coupling and bending stiffness matrices, i.e. A,

B and D defined as per classical laminated plate theory for thin plates:

$$U_{2D} = \frac{1}{2}\begin{bmatrix}\epsilon & \rho\end{bmatrix}\begin{bmatrix}A & B\\ B & D\end{bmatrix}\begin{bmatrix}\epsilon\\ \rho\end{bmatrix} \tag{A.8}$$

Now, the energy functional as given by Eq. A.8, is to be minimized with respect the warping variables. However from the zeroth order expressions for ϵ_{22}, $2\epsilon_{12}$ and ρ_{22} . It can be deduced that minimization with respect to the warping variables w_1, w_2 and w_3 is equivalent to a minimization with respect to the membrane strains and curvature ϵ_{22}, $2\epsilon_{12}$ and ρ_{22} respectively [43]. After minimization, the membrane strains ϵ_{22} , $2\epsilon_{12}$ and ρ_{22} are obtained in terms of ϵ_{11} and ρ_{12}, thereafter the two dimensional strain energy, i.e. U_{2D} is integrated along the length of the strip to yield an expression for one dimensional strain energy, i.e. U_{1D} in terms of the strain measures, i.e. γ_{11}, κ_1, κ_2 and κ_3 along the beam reference line. The coefficients for each of the one-dimensional strain measures are then interpreted as terms of the cross-sectional stiffness matrix. i.e.

$$U_{1D} = \frac{1}{2}\epsilon_l^T[S_l]\epsilon_l + \epsilon_l^T[S_{ln}]\epsilon_n + \frac{1}{2}\epsilon_n^T[S_n]\epsilon_n$$

where,

$$\begin{aligned}\epsilon_l &= [\gamma_{11} \quad \kappa_1 \quad \kappa_2 \quad \kappa_3]\\ \epsilon_n &= [\kappa_1^2 \quad \kappa_2^2 \quad \kappa_2\gamma_{11} \quad \kappa_2\kappa_3 \quad \kappa_2\kappa_1]\end{aligned}$$

For a strip with isotropic material, with Young's modulus E, Poisson's ratio v and pretwist along the reference line k_1, the stiffness matrices S_l, S_{ln} and S_n are:

$$S_l = \begin{bmatrix} Ebt & \frac{Eb^3tk_1}{12} & 0 & 0\\ \frac{Eb^3tk_1}{12} & \frac{Et^3b}{6(1+v)} + \frac{Eb^5tk_1^2}{80} & 0 & 0\\ 0 & 0 & \frac{Et^3b}{12} & 0\\ 0 & 0 & 0 & \frac{Eb^3t}{12}\end{bmatrix} \tag{A.9}$$

$$S_{ln} = \begin{bmatrix} \frac{Eb^3t}{24} & 0 & 0 & 0 & 0\\ \frac{Eb^5tk_1}{160} & \frac{Eb^5tvk_1}{360(1-v^2)} & 0 & 0 & 0\\ 0 & 0 & 0 & 0 & 0\\ 0 & 0 & 0 & 0 & 0\end{bmatrix} \tag{A.10}$$

$$S_n = \begin{bmatrix} \frac{Eb^5t}{320} & 0 & 0 & 0 & 0 \\ 0 & \frac{Eb^5\nu^2t}{720} & 0 & 0 & 0 \\ 0 & 0 & 0 & 0 & 0 \\ 0 & 0 & 0 & -\frac{Eb^7}{2520t} & 0 \\ 0 & 0 & 0 & 0 & \frac{Eb^5t\nu}{360} - \frac{Eb^9k_1^2(1-\nu^2)}{7560t} \end{bmatrix} \tag{A.11}$$

B. Tubular geometry

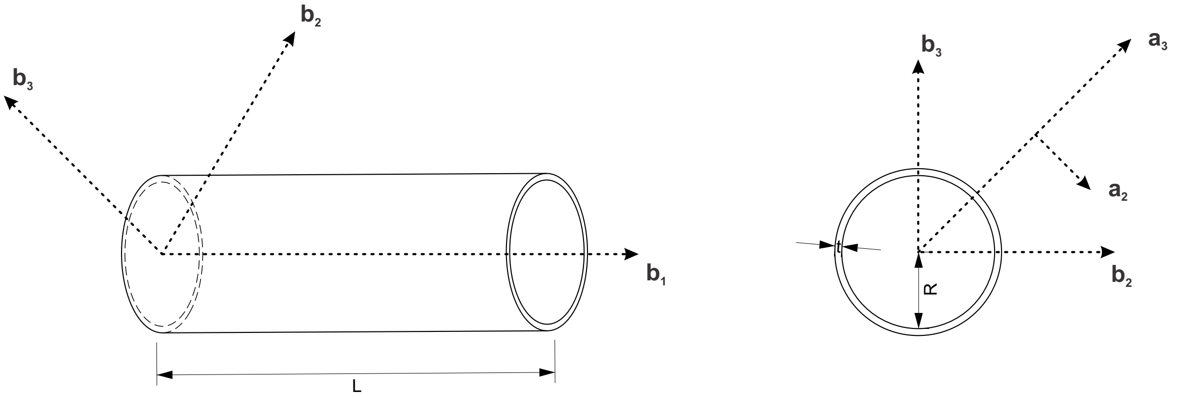

Figure B1: Thin circular tube with geometric parameters : length L, radius R and thickness t. Position vector to a material point on the undeformed cross-section, i.e. $\hat{\boldsymbol{r}}$ is expressed in terms of measure numbers along the orthonormal triad of $\boldsymbol{b_i}$ vectors. For the curvilinear coordinate system is $\boldsymbol{a}_1$ coincides with $\boldsymbol{b}_1$ and $\boldsymbol{a}_2$ and $\boldsymbol{a}_3$ are along the tangential and radial directions respectively.

For the circular tube, in addition to the orthonormal triad of $\boldsymbol{b_i}$ vectors described in Appendix A for the strip, it is necessary to introduce a curvilinear coordinate system as shown in Fig. B1 such that the $\boldsymbol{a_1}$ coincides with $\boldsymbol{b_1}$ vector, while $\boldsymbol{a_2}$ and $\boldsymbol{a_3}$ vectors are along the tangential and radial directions respectively. The position vector, i.e. $\hat{\boldsymbol{r}}$ for any point on the cross-section of the undeformed beam can then be expressed as follows[44]:

$$\hat{\boldsymbol{r}} = x_1\boldsymbol{b_1} + x_2\boldsymbol{b_2}(x_1) + x_3\boldsymbol{b_3}(x_1) = x_1\boldsymbol{a_1} + (R + y_3)\boldsymbol{a_2}(y_2)$$

Similarly, a curvilinear coordinate system $\boldsymbol{A_i}$ is introduced for the deformed configuration as well,

such that the orthonormal triad of basis vectors $\boldsymbol{B_i}$ and $\boldsymbol{A_i}$ can be related as follows:

$$\boldsymbol{B_1} = \boldsymbol{A_1}$$
$$\boldsymbol{B_2} = cos\left(\frac{y_2}{R}\right)\boldsymbol{A_2} + sin\left(\frac{y_2}{R}\right)\boldsymbol{A_3}$$
$$\boldsymbol{B_3} = cos\left(\frac{y_2}{R}\right)\boldsymbol{A_3} - sin\left(\frac{y_2}{R}\right)\boldsymbol{A_2}$$

The position of a material point on the deformed cross-section, i.e. $\hat{\boldsymbol{R}}$ is expressed as follows:

$$\hat{\boldsymbol{R}} = y_1\boldsymbol{b_1} + u_i\boldsymbol{b_i} + (R + y_3)\boldsymbol{A_3} + w_i\boldsymbol{A_i} \tag{B.1}$$

The deformation gradient tensor is determined using the definition of covariant and contra-variant vectors as described for the strip in Appendix A. The three-dimensional strains are then determined in accordance with the following definition for Green's strain [44]:

$$\Gamma = \frac{X^TX - I}{2} \tag{B.2}$$

where, I is the identity matrix. The methodology for minimization of the strain energy functional for the circular tube is similar to the formulations adopted for the strip [44].

C. Geometrically exact beam theory

The equations for one-dimensional beam analysis are derived from extended Hamilton's principle[49]:

$$\int_{t_1}^{t_2}\int_0^L [\delta(T - U) + \delta\bar{W}] = \delta\bar{A} \tag{C.1}$$

where T is the kinetic energy per unit length, U is the strain energy per unit length W is the virtual work done by the applied loads per unit length, A is the virtual action at the ends of the beam of length L, and t_1 and t_2, are arbitrary instances of time [49]. For a static problem, the kinetic energy term is eliminated. The variation of the strain energy, which is a function of one-dimensional force and moment strains along the beam reference line, i.e. γ and κ can be written as follows:

$$\int_0^L \delta U dx_1 = \int_0^L \left[\delta\gamma^T\left(\frac{\partial U}{\partial\gamma}\right) + \delta\kappa^T\left(\frac{\partial U}{\partial\kappa}\right)\right]dx_1 \tag{C.2}$$

The partial derivative of the one-dimensional strain energy can be recognized as sectional force resultants, i.e:

$$F = \left(\frac{\partial U}{\partial \gamma}\right) \quad M = \left(\frac{\partial U}{\partial \kappa}\right) \tag{C.3}$$

where, F is the column matrix with axial and shear forces and M is the column matrix with twisting and bending moments. Further, the variation of strain measures along the beam reference line are expressed as functions of virtual displacement and rotations, i.e. $\overline{\delta q}$ and $\overline{\delta \psi}$:

$$\delta\kappa = \overline{\delta\psi}' + \tilde{K}\overline{\delta\psi} \tag{C.4}$$

$$\delta\gamma = \overline{\delta q}' + \tilde{K}\overline{\delta q} + (\tilde{\gamma} + \tilde{e}_1)\overline{\delta\Psi} \tag{C.5}$$

where, K is the curvature vector for the reference line of the deformed beam. Substituting the expressions for variation of strain measures in Eq. C.2, the variation of strain energy can be written as follows:

$$\begin{aligned}\int_0^L \delta U dx_1 &= \int_0^L \delta\gamma^T F + \delta^T M dx_1 \\ &= \int_0^L \left[(\overline{\delta q'})^T - \overline{\delta q}^T \tilde{K} - \overline{\delta\Psi}^T(\tilde{\gamma} + \tilde{e}_1)\right]F + \left[(\overline{\delta\psi'})^T - \overline{\delta\psi}^T \tilde{K}\right]M dx_1\end{aligned}$$

Substituting the above equation in Eq. C.1 :

$$\int_{t_1}^{t_2}\int_0^l \delta U - \delta W dx_1 dt = \int_{t_1}^{t_2}\int_0^l \left[(\overline{\delta q'})^T - \overline{\delta q}^T \tilde{K} - \overline{\delta\Psi}^T(\tilde{\gamma} + \tilde{e}_1)\right]F + \left[(\overline{\delta\psi'})^T - \overline{\delta\psi}^T \tilde{K}\right]M \quad (*)$$

$$- (\overline{\delta q})^T f - (\overline{\delta\psi})^T m dx_1 dt = \int_{t_1}^{t_2} (\overline{\delta q})^T \hat{F} + (\overline{\delta\psi})^T \hat{M}|_0^L dt \tag{C.6}$$

Eq. C.6 is integrated by parts to get the following Euler-Lagrange's equations:

$$F' + \tilde{K}F + f = 0$$

$$M + \tilde{K}M + m + (\tilde{\gamma} + \tilde{e}_1)F = 0$$

The above equations are used in conjunctions with kinematical equations and constitutive relations to determine the complete set of variables. Hodges [47] presents the following mixed variational formulation for the one-dimensional beam equations, wherein the kinematical equations are added to the variational formulation. This involves constrained minimization of Hamilton's integral and

determining the associated Lagrange's parameters:

$$\int_{t_1}^{t_2}\int_0^L\Big\{\Big[(\overline{\delta q'})^T-\overline{\delta q}^T\tilde{K}-\overline{\delta\Psi}^T(\tilde{\gamma}+\tilde{e_1})\Big]F+\Big[(\overline{\delta\psi'})^T-\overline{\delta\psi}^T\tilde{K}\Big]M+\delta\gamma^T\Big[\Big(\frac{\partial U}{\partial\gamma}\Big)^T-F\Big]+\delta\kappa^T\Big[\Big(\frac{\partial U}{\partial\kappa}\Big)^T-M\Big]$$
$$+\overline{\delta F}^T[e_1+\tilde{k}u-C^T(e_1+\gamma)]-\Big(\overline{\delta F}'\Big)^T u+\overline{\delta M}^T\Big[\Delta+\frac{\tilde{\theta}}{2}+\frac{\theta\theta^T}{4}\Big](Ck-k-\kappa)$$
$$-\Big(\overline{\delta M}'\Big)^T\theta-(\overline{\delta q})^T f-(\overline{\delta\psi})^T m\Big\}dx_1dt=\int_{t_1}^{t_2}(\overline{\delta q})^T\hat{F}+(\overline{\delta\psi})^T\hat{M}-\Big(\overline{\delta F}\Big)^T\hat{u}-\Big(\overline{\delta M}\Big)^T\hat{\theta}\Big|_0^L dt$$

where, C is the rotation matrix expressed in terms of the Rodrigues parameter θ [47]:

$$C=\frac{(1-\frac{1}{4}\theta^T\theta)\Delta-\tilde{\theta}+\frac{1}{2}\theta\theta^T}{(1+\frac{1}{4}\theta^T\theta)} \tag{C.7}$$

W.Yu and M.Blair [49] present a general purpose finite element implementation for the above equation, wherein the following system of equations is solved for the discretized beam with N elements and N+1 nodes:
At the starting node:

$$f_{u_1}^- - F_1^\star=0 \quad f_{\psi_1}^- - M_1^\star=0 \quad f_{F_1}^- - \hat{u}_1=0 \quad f_{M_1}^- - \hat{\theta}_1=0 \tag{C.8}$$

At the ending node:

$$f_{u_N}^+ - F_{N+1}^\star=0 \quad f_{\psi_N}^+ - M_{N+1}^\star=0 \quad f_{F_N}^+ - \hat{u}_{N+1}=0 \quad f_{M_N}^+ - \hat{\theta}_{N+1}=0 \tag{C.9}$$

At the intermediate node:

$$f_{u_i}^+ + f_{u_{i+1}}^-=0 \quad f_{\psi_i}^+ + f_{\psi_{i+1}}^-=0 \quad f_{F_i}^+ + f_{F_{i+1}}^-=0 \quad f_{M_i}^+ + f_{M_{i+1}}^-=0 \tag{C.10}$$

where,

$$f_{u_i}^\pm=\pm C^TC^{ab}F_i-\bar{f}_i^\pm \tag{C.11}$$

$$f_{\psi_i}^\pm=\pm C^TC^{ab}M_i-\bar{m}_i^\pm-\frac{\Delta L_i}{2}\Big[(\tilde{e}_1+\tilde{\gamma}_1)F_i\Big] \tag{C.12}$$

$$f_{F_i}^\pm=\pm u_i-\frac{\Delta L_i}{2}\Big[C^TC^{ab}(e_1+\gamma_1)-C^{ab}e_1\Big] \tag{C.13}$$

$$f_{M_i}^\pm=\pm\theta_i-\frac{\Delta L_i}{2}\Big[\Delta+\frac{\tilde{\theta}_i}{2}+\frac{\tilde{\theta}_i\tilde{\theta}_i^T}{4}\Big]C^{ab}\kappa_i \tag{C.14}$$

$$\bar{f}_i^- = \int_0^1 (1-\epsilon) f_a \Delta L_i d\epsilon \qquad \bar{f}_i^+ = \int_0^1 \epsilon f_a \Delta L_i d\epsilon \tag{C.15}$$

$$\bar{m}_i^- = \int_0^1 (1-\epsilon) m_a \Delta L_i d\epsilon \qquad \bar{m}_i^+ = \int_0^1 \epsilon m_a \Delta L_i d\epsilon \tag{C.16}$$

The one-dimensional strains along the beam reference line are determined using the following equations:

$$\begin{bmatrix} \gamma \\ \kappa \end{bmatrix} = S \begin{bmatrix} F \\ M \end{bmatrix} \tag{C.17}$$
